# Supplementary material for: Effectiveness and protection duration of Covid-19 vaccines and previous infection against any SARS-CoV-2 infection in young adults
Source: Nat Commun. 2022 Jul 8;13:3946. doi: 10.1038/s41467-022-31469-z (PMC9263799; doi:10.1038/s41467-022-31469-z)
Supplement: Supplementary file 1 — Supplementary Information [file 41467_2022_31469_MOESM1_ESM.pdf]

Title: Effectiveness and Protection Duration of Covid-19 Vaccines and Previous Infection Against Any SARS-CoV-2 Infection

Authors: Lior Rennert,[1*] PhD, Zichen Ma,[1] PhD, Christopher S. McMahan,[2] PhD, Delphine Dean,[3] PhD

Affiliations
[1] Department of Public Health Sciences, Clemson University, Clemson, SC, USA
[2] School of Mathematical and Statistical Sciences, Clemson University, Clemson, SC, USA
[3] Department of Bioengineering, Clemson University, Clemson, SC, USA

***Corresponding author**
Lior Rennert, PhD
Department of Public Health Sciences, Clemson University
517 Edwards Hall
Clemson, SC, 29601
864-656-7434
liorr@clemson.edu

Table of Contents

Supplementary Note 1. Statistical models for overall protection from vaccination and previous infection (Table 2) 3

Supplementary Note 2. Statistical models for waning protection from vaccination and previous infection (Table 3) 7

Table S1 9

Table S2 10

Table S3 11

Table S4 12

Table S5 13

Table S6 14

Table S7 15

Table S8 16

Table S9 17

Table S10 18

Table S11 19

Table S12 20

Table S13 21

Figure S1 22

Figure S2 23

Figure S3 24

Supplementary Note 1. Statistical models for overall protection from vaccination and previous infection (Table 2)

Vaccine protection (across manufacturers):

$$h(t|V_i, P_i, \boldsymbol{X}_i) = h_0(t) \times \exp\{\alpha \times V_{Pi}(t) + \beta \times V_{Ci}(t) + \nu \times P_i + \boldsymbol{\eta}'\boldsymbol{X}_i\} \quad \text{(Model 1.1)}$$

- Protection from full vaccination: $1 - \exp\{\beta\}$

where

- $V_{Pi}(t)$ = 1 if subject i is partially vaccinated at time *t*, and 0 otherwise (partially vaccinated = 14 days past first dose of mRNA-1273 or BNT162b2 and without second dose by time *t*)
- $V_{C_i}(t)$ = 1 if subject i is fully vaccinated at time *t*, and 0 otherwise (fully vaccinated = 14 days past second dose of mRNA-1273 or BNT162b2, or 14 days past their first dose of Ad26.COV2.S)
- P_i=1 if subject *i* has previous SARS-CoV-2 infection occurring prior to the follow-up period, and 0 otherwise.
    - Note that P_i remains time-invariant in this setting since it is not possible to have a previous infection occur during the follow-up period (since this is the event of interest and would therefore be classified as an infection during follow-up).

$\boldsymbol{X}_i$ = covariate vector for subject i, and includes age, race/ethnicity (categories defined in Table 1), gender (categories defined in Table 1), residential status, self-reported presence of any of the following conditions: high blood pressure, heart disease, diabetes, overweight or obesity, kidney disease or dialysis, previous stroke or other neurological condition affecting my ability to cough, liver disease, or lung disease, self-reported presence of any condition impacting immune response: HIV, Cancer, Lupus, Rheumatoid Arthritis, Solid organ or bone marrow transplant, self-reported medication use of any of the following: steroids, chemotherapy, immunosuppressants, and self-reported use of tobacco or nicotine products.

Vaccine protection (by manufacturer)

$$h(t|\boldsymbol{V}_i, P_i, \boldsymbol{X}_i) = h_0(t) \times \exp\{\sum_{k=1:2} \alpha_k \times V_{P_k,i}(t) + \sum_{l=1:3} \beta_l \times V_{C_l,i}(t) + \nu \times P_i + \boldsymbol{\eta}'\boldsymbol{X}_i\} \quad \text{(Model 1.2)}$$

- Protection from full vaccination by mRNA-1273: $1 - \exp\{\beta_1\}$
- Protection from full vaccination by BNT162b2: $1 - \exp\{\beta_2\}$
- Protection from full vaccination by Ad26.COV2.S: $1 - \exp\{\beta_3\}$

where

- $V_{P_1,i}(t) = 1$ if subject *i* is partially vaccinated from mRNA-1273 at time *t*, and 0 otherwise
- $V_{P_2,i}(t) = 1$ if subject *i* is partially vaccinated from BNT162b2 at time *t*, and 0 otherwise
- $V_{C_1,i}(t) = 1$ if subject *i* is fully vaccinated from mRNA-1273 at time *t*, and 0 otherwise
- $V_{C_2,i}(t) = 1$ if subject *i* is fully vaccinated from BNT162b2 at time *t*, and 0 otherwise
- $V_{C_3,i}(t) = 1$ if subject *i* is fully vaccinated from Ad26.COV2.S at time *t*, and 0 otherwise

Differences in vaccine effectiveness between mRNA-1273 and BNT162b2 is evaluated by testing the null hypothesis $H_0: \beta_1 = \beta_2$. Differences in vaccine effectiveness between mRNA-1273 and Ad26.COV2.S is evaluated by testing the null hypothesis $H_0: \beta_1 = \beta_3$. Differences in vaccine effectiveness between BNT162b2 and Ad26.COV2.S is evaluated by testing the null hypothesis $H_0: \beta_2 = \beta_3$.

Vaccine protection by previous SARS-CoV-2 infection history (across manufacturers)

$$h(t|V_i, P_i, \boldsymbol{X}_i) = h_0(t) \times \exp\left\{\begin{matrix} \alpha \times V_{Pi}(t) + \beta \times V_{Ci}(t) + \nu \times P_i + \gamma_1 \times V_{P_i}(t) \times P_i \\ +\gamma_2 \times V_{Ci}(t) \times P_i + \boldsymbol{\eta}'\boldsymbol{X}_i \end{matrix}\right\} \quad \text{(Model 1.3)}$$

- Protection from full vaccination without previous SARS-CoV-2 infection: $1 - \exp\{\beta\}$
- Protection from previous infection only: $1 - \exp\{\nu\}$
- Protection from full vaccination with previous SARS-CoV-2 infection: $1 - \exp\{\beta + \nu + \gamma_2\}$

Vaccine protection by previous SARS-CoV-2 infection history (by manufacturer)

$$h(t|V_i, P_i, \boldsymbol{X}_i) = h_0(t) \times \exp\left\{\begin{matrix} \sum_{k=1:2} \alpha_k \times V_{P_k,i}(t) + \sum_{l=1:3} \beta_l \times V_{C_l,i}(t) + \nu \times P_i + \\ \sum_{k=1:2} \gamma_{1k} \times V_{P_k,i}(t) \times P_i + \sum_{l=1:3} \gamma_{2l} \times V_{C_l,i}(t) \times P_i + \boldsymbol{\eta}'\boldsymbol{X}_i \end{matrix}\right\} \quad \text{(Model 1.4)}$$

- Protection from full vaccination by mRNA-1273 without previous SARS-CoV-2 infection: $1 - \exp\{\beta_1\}$
- Protection from full vaccination by BNT162b2 without previous SARS-CoV-2 infection: $1 - \exp\{\beta_2\}$
- Protection from full vaccination by Ad26.COV2.S without previous SARS-CoV-2 infection: $1 - \exp\{\beta_3\}$
- Protection from full vaccination by mRNA-1273 with previous SARS-CoV-2 infection: $1 - \exp\{\beta_1 + \nu + \gamma_{21}\}$
- Protection from full vaccination by BNT162b2 with previous SARS-CoV-2 infection: $1 - \exp\{\beta_2 + \nu + \gamma_{22}\}$
- Protection from full vaccination by Ad26.COV2.S with previous SARS-CoV-2 infection: $1 - \exp\{\beta_3 + \nu + \gamma_{23}\}$

Protection from vaccination adjusted for time since previous infection (across manufacturers)

$h(t|V_i, P_i, \boldsymbol{X}_i) = h_0(t) \times \exp\{\alpha \times V_{Pi}(t) + \beta \times V_{Ci}(t) + \nu_1 \times P_i + \nu_2 \times P_i \times T_{Pi}(t) + \boldsymbol{\eta}'\boldsymbol{X}_i\}$ (Model 1.5)

- Protection from full vaccination: $1 - \exp\{\beta\}$

where

- $V_{Pi}(t)$ = 1 if subject *i* is partially vaccinated at time *t*, and 0 otherwise (partially vaccinated = 14 days past first dose of mRNA-1273 or BNT162b2 and without second dose by time *t*)
- $V_{C_i}(t)$ = 1 if subject *i* is fully vaccinated at time *t*, and 0 otherwise (fully vaccinated = 14 days past second dose of mRNA-1273 or BNT162b2, or 14 days past their first dose of Ad26.COV2.S)
- P_i=1 if subject *i* has previous SARS-CoV-2 infection occurring prior to the follow-up period, and 0 otherwise.
    - Note that P_i remains time-invariant in this setting since it is not possible to have a previous infection occur during the follow-up period (since this is the event of interest and would therefore be classified as an infection during follow-up).
- $T_{Pi}(t)$ is days between date of previous SARS-CoV-2 infection and time *t*

Protection from vaccination adjusted for time since previous infection (by manufacturer)

$$h(t|\boldsymbol{V}_i, P_i, \boldsymbol{X}_i) = h_0(t) \times \exp\left\{\begin{matrix} \sum_{k=1:2} \alpha_k \times V_{P_k,i}(t) + \sum_{l=1:3} \beta_l \times V_{C_l,i}(t) + \nu_1 \times P_i + \\ \nu_2 \times P_i \times T_{Pi}(t) + \boldsymbol{\eta}'\boldsymbol{X}_i \end{matrix}\right\} \quad \text{(Model 1.6)}$$

- Protection from full vaccination by mRNA-1273: $1 - \exp\{\beta_1\}$
- Protection from full vaccination by BNT162b2: $1 - \exp\{\beta_2\}$
- Protection from full vaccination by Ad26.COV2.S: $1 - \exp\{\beta_3\}$

where

- $V_{P_1,i}(t)$ = 1 if subject *i* is partially vaccinated from mRNA-1273 at time *t*, and 0 otherwise
- $V_{P_2,i}(t)$ = 1 if subject *i* is partially vaccinated from BNT162b2 at time *t*, and 0 otherwise
- $V_{C_1,i}(t)$ = 1 if subject *i* is fully vaccinated from mRNA-1273 at time *t*, and 0 otherwise
- $V_{C_2,i}(t)$ = 1 if subject *i* is fully vaccinated from BNT162b2 at time *t*, and 0 otherwise
- $V_{C_3,i}(t)$ = 1 if subject *i* is fully vaccinated from Ad26.COV2.S at time *t*, and 0 otherwise

Protection from vaccination and previous infection, adjusted for time since vaccination (across manufacturers)

$$h(t|V_i, P_i, \boldsymbol{X}_i) = h_0(t) \times \exp\left\{\begin{matrix} \alpha_1 \times V_{Pi}(t) + \alpha_2 \times V_{Pi}(t) \times T_{V_{pi}}(t) + \beta_1 \times V_{Ci}(t) + \beta_2 \times T_{V_{Ci}}(t) + \\ \nu \times P_i + \gamma_1 \times V_{P_i}(t) \times P_i + \gamma_2 \times V_{Ci}(t) \times P_i + \boldsymbol{\eta}'\boldsymbol{X}_i \end{matrix}\right\} \quad \text{(Model 1.7)}$$

- Protection from previous SARS-CoV-2 infection only: $1 - \exp\{\nu\}$
- HR of full vaccination with previous SARS-CoV-2 infection against full vaccination without previous SARS-CoV-2 infection: $\exp\{\nu + \gamma_2\}$

Protection from vaccination and previous infection, adjusted for time since vaccination (by manufacturer)

$$h(t|\boldsymbol{V}_i, P_i, \boldsymbol{X}_i) = h_0(t) \times \exp \left\{ \begin{array}{c} \sum_{k=1:2} \alpha_{1k} \times V_{P_k,i}(t) + \sum_{k=1:2} \alpha_{2k} \times V_{P_k,i}(t) \times T_{V_{P_i,k}}(t) + \\ \sum_{l=1:3} \beta_{1l} \times V_{C_l,i}(t) + \sum_{l=1:3} \beta_{2l} \times V_{C_l,i}(t) \times T_{V_{C_i,k}}(t) + \nu_1 \times P_i + \\ \sum_{k=1:2} \nu_{2k} \times V_{P_k,i}(t) \times P_i + \sum_{l=1:3} \nu_{3l} \times V_{C_l,i}(t) \times P_i + \boldsymbol{\eta}'\boldsymbol{X}_i \end{array} \right\} \text{(Model 1.8)}$$

- HR of full mRNA-1273 vaccination with previous SARS-CoV-2 infection against full mRNA-1273 vaccination without previous SARS-CoV-2 infection: exp $\{\nu_1 + \nu_{31}\}$
- HR of full BNT162b2 vaccination with previous SARS-CoV-2 infection against full BNT162b2 vaccination without previous SARS-CoV-2 infection: exp $\{\nu_1 + \nu_{32}\}$
- HR of full Ad26.COV2.S vaccination with previous SARS-CoV-2 infection against full Ad26.COV2.S vaccination without previous SARS-CoV-2 infection: exp $\{\nu_1 + \nu_{33}\}$

Supplementary Note 2. Statistical models for waning protection from vaccination and previous infection (Table 3)

Vaccine protection (across manufacturers):

$$h(t|V_i, P_i, \boldsymbol{X}_i) = h_0(t) \times \exp\begin{Bmatrix} \alpha_1 \times V_{Pi}(t) + \alpha_2 \times V_{Pi}(t) \times T_{V_{P_i}}(t) + \beta_1 \times V_{Ci}(t) + \\ \beta_2 \times V_{Ci}(t) \times T_{V_{C_i}}(t) + \nu_1 \times P_i + \nu_2 \times P_i \times T_{P_i}(t) + \boldsymbol{\eta}'\boldsymbol{X}_i \end{Bmatrix} \quad \text{(Model 2.1)}$$

- Protection from full vaccination at 3 months: $1 - \exp\{\beta_1 + \beta_2 \times 90\}$
- Protection from full vaccination at 6 months: $1 - \exp\{\beta_1 + \beta_2 \times 180\}$
  - Hazard ratio for change in monthly risk: $\exp\{\beta_2 \times 30\}$
- Protection from previous infection at 3 months: $1 - \exp\{\nu_1 + \nu_2 \times 90\}$
- Protection from previous infection at 6 months: $1 - \exp\{\nu_1 + \nu_2 \times 180\}$
  - Hazard ratio for change in monthly risk: $\exp\{\nu_2 \times 30\}$

where

- $T_{V_{P_i}}$ is days between date of partial vaccination and time *t*
- $T_{V_{C_i}}$ is days between date of full vaccination and time *t*
- T_{P_i} is days between date of previous SARS-CoV-2 infection and time *t*

Vaccine protection (by manufacturer)

$$h(t|\boldsymbol{V}_i, P_i, \boldsymbol{X}_i) = h_0(t) \times \exp\begin{Bmatrix} \sum_{k=1:2} \alpha_{1k} \times V_{P_k,i}(t) + \sum_{k=1:2} \alpha_{2k} \times V_{P_k,i}(t) \times T_{V_{P_{i,k}}}(t) + \\ \sum_{l=1:3} \beta_{1l} \times V_{C_l,i}(t) + \sum_{l=1:3} \beta_{2l} \times V_{C_l,i}(t) \times T_{V_{C_{i,k}}}(t) + \\ \nu_1 \times P_i + \nu_2 \times P_i \times T_{P_i}(t) + \boldsymbol{\eta}'\boldsymbol{X}_i \end{Bmatrix} \quad \text{(Model 2.2)}$$

- Protection from full vaccination by mRNA-1273 at 3 months: $1 - \exp\{\beta_{11} + \beta_{21} \times 90\}$
- Protection from full vaccination by mRNA-1273 at 6 months: $1 - \exp\{\beta_{11} + \beta_{21} \times 180\}$
  - Hazard ratio for change in monthly risk: $\exp\{\beta_{21} \times 30\}$
- Protection from full vaccination by BNT162b2 at 3 months: $1 - \exp\{\beta_{12} + \beta_{22} \times 90\}$
- Protection from full vaccination by BNT162b2 at 6 months: $1 - \exp\{\beta_{12} + \beta_{22} \times 180\}$
  - Hazard ratio for change in monthly risk: $\exp\{\beta_{22} \times 30\}$
- Protection from full vaccination by Ad26.COV2.S at 3 months: $1 - \exp\{\beta_{13} + \beta_{23} \times 90\}$
- Protection from full vaccination by Ad26.COV2.S at 6 months: $1 - \exp\{\beta_{13} + \beta_{23} \times 180\}$
  - Hazard ratio for change in monthly risk: $\exp\{\beta_{23} \times 30\}$

where

- $T_{V_{P_{1,k}}}$ is days between date of first dose of mRNA-1273 and time *t*
- $T_{V_{P_{2,k}}}$ is days between date of first dose of BNT162b2 and time *t*
- $T_{V_{C_{1,k}}}$ is days between date of second dose of mRNA-1273 and time *t*
- $T_{V_{C_{2,k}}}$ is days between date of second dose of BNT162b2 and time *t*
- $T_{V_{C_{3,k}}}$ is days between date of first dose of Ad26.COV2.S and time *t*

Difference in protection at 6 months between mRNA-1273 and BNT162b2 is evaluated by testing the null hypothesis H_0: $\beta_{11} + \beta_{21} \times 180 = \beta_{12} + \beta_{22} \times 180$. Approximate distribution of $\left(\hat{\beta}_{11} - \hat{\beta}_{12}\right) + \left(\hat{\beta}_{21} - \hat{\beta}_{22}\right) \times 180$ is derived using the multivariate delta method, assuming the joint distribution of $(\hat{\beta}_{11}, \hat{\beta}_{12}, \hat{\beta}_{21}, \hat{\beta}_{22})$ is approximately normal. Similarly, difference in protection at 6 months between mRNA-1273 and Ad26.COV2.S is evaluated by testing the null hypothesis H_0: $\beta_{11} + \beta_{21} \times 180 = \beta_{13} + \beta_{23} \times 180$. Difference in protection at 6 months between BNT162b2 and Ad26.COV2.S is evaluated by testing the null hypothesis H_0: $\beta_{12} + \beta_{22} \times 180 = \beta_{13} + \beta_{23} \times 180$.

Table S1: Descriptive characteristics for main study sample by vaccine manufacturer

**Characteristic**	**Fully vaccinated N = 12786**	**mRNA-1273 N = 4562**	**BNT162b2 N = 7276**	**Ad26.COV2.S N = 948**	**P-value**
Age: Mean (SD)	20.03 (1.56)	20.41 (1.54)	19.77 (1.53)	20.14 (1.52)	<0.001*
Race/Ethnicity: N (%)					0.001**
...White, non-Hispanic	10023(78.4%)	3568(78.2%)	5662(77.8%)	793(83.6%)	<0.001**
...Black, non-Hispanic	824(6.4%)	312(6.8%)	478(6.6%)	34(3.6%)	<0.001**
...Any race, Hispanic	857(6.7%)	301(6.6%)	507(7.0%)	49(5.2%)	0.10**
...All other, races non-Hispanic	1082(8.5%)	381(8.4%)	629(8.6%)	72(7.6%)	0.54**
Gender: N (%)					<0.001**
...Female	7153(55.9%)	2506(54.9%)	4288(58.9%)	359(37.9%)	<0.001**
...Male	5607(43.9%)	2046(44.8%)	2974(40.9%)	587(61.9%)	<0.001**
...Not reported	26(0.2%)	10(0.2%)	14(0.2%)	2(0.2%)	0.86**
Affiliation: N (%)					<0.001**
...Residential	4415(34.5%)	1100(24.1%)	3027(41.6%)	288(30.4%)	<0.001**
...Non-residential	8371(65.5%)	3462(75.9%)	4249(58.4%)	660(69.6%)	<0.001**
**Condition impacting immune response: N (%)♦**	238(1.9%)	92(2.0%)	121(1.7%)	25(2.6%)	0.07**
...Lung disease	74(0.6%)	35(0.8%)	36(0.5%)	3(0.3%)	0.11**
...HIV	3(0.0%)	2(0.0%)	0(0.0%)	1(0.1%)	0.04**
...Cancer	9(0.1%)	4(0.1%)	5(0.1%)	0(0.0%)	0.87**
...Lupus	5(0.0%)	2(0.0%)	2(0.0%)	1(0.1%)	0.33**
...Rheumatoid arthritis	17(0.1%)	7(0.2%)	9(0.1%)	1(0.1%)	0.93**
...Solid organ or bone marrow transplant	10(0.1%)	5(0.1%)	4(0.1%)	1(0.1%)	0.38**
**Any other pre-existing condition: N (%)▪**	731(5.7%)	301(6.6%)	375(5.2%)	55(5.8%)	0.005**
...High blood pressure	88(0.7%)	44(1.0%)	36(0.5%)	8(0.8%)	0.009**
...Heart disease	17(0.1%)	7(0.2%)	10(0.1%)	0(0.0%)	0.74**
...Diabetes	72(0.6%)	26(0.6%)	42(0.6%)	4(0.4%)	0.93**
...Overweight	374(2.9%)	160(3.5%)	186(2.6%)	28(3.0%)	0.01**
...Kidney disease	12(0.1%)	4(0.1%)	7(0.1%)	1(0.1%)	1.00**
...Cough inefficacy	4(0.0%)	1(0.0%)	3(0.0%)	0(0.0%)	1.00**
...Liver disease	8(0.1%)	1(0.0%)	7(0.1%)	0(0.0%)	0.29**
**Medications▲ N (%)**	306(2.4%)	117(2.6%)	168(2.3%)	21(2.2%)	0.64**
...Steroids	67(0.5%)	26(0.6%)	40(0.5%)	1(0.1%)	0.15**
...Chemotherapy	4(0.0%)	1(0.0%)	3(0.0%)	0(0.0%)	1.00**
...Immunosuppressants	73(0.6%)	29(0.6%)	39(0.5%)	5(0.5%)	0.78**
**Use of tobacco or nicotine products: N (%)**	576(4.5%)	249(5.5%)	268(3.7%)	59(6.2%)	<0.001**
**SARS-CoV-2 Tests Per Person: Mean (SD)**	26.15 (11.89)	28.18 (11.81)	25.13 (11.82)	24.31 (11.42)	<0.001*
...Fall 2020 Semester	5.42 (3.35)	5.42 (3.37)	5.49 (3.34)	4.93 (3.23)	<0.001*
...Spring 2021 Semester	11.36 (5.53)	11.49 (5.46)	11.38 (5.53)	10.47 (5.73)	<0.001*
...Fall 2021 Semester¶	14.21 (3.89)	14.34 (3.77)	14.23 (3.91)	13.35 (4.14)	<0.001*
**Previous SARS-CoV-2 Infection: N (%)†**	2629(20.6%)	930(20.4%)	1471(20.2%)	228(24.1%)	0.02**
**SARS-CoV-2 Infections During Follow-up: N (%)#**	518(4.1%)	131(2.9%)	325(4.5%)	62(6.5%)	<0.001**

♦ Self-reported presence of any condition impacting immune response: HIV, Cancer, Lupus, Rheumatoid Arthritis, Solid organ or bone marrow transplant; sample size may not add to N due to non-selection of specific conditions.

▪ Self-reported presence of any of the following conditions: high blood pressure, heart disease, diabetes, overweight or obesity, kidney disease or dialysis, previous stroke or other neurological condition affecting my ability to cough, liver disease, or lung disease; sample size may not add to N due to non-selection of specific conditions.

▲ Self-reported medication use of any of the following: steroids, chemotherapy, immunosuppressants; sample size may not add to N due to non-selection of specific medications.

¶ During follow-up period (8/8/2021 to 12/4/2021)

† Infection occurring prior to follow-up period (8/7/21)

% is proportion of individuals within each population infected with SARS-CoV-2 during follow-up period

* Based on ANOVA F-test

** Based on chi-square test for independence

Table S2: Descriptive characteristics for study sample between August 8th, 2021 and December 4th, 2021, restricted to individuals with a SARS-CoV-2 testing history prior to November 25th, 2020.

**Characteristic**	**Total**<br>**N = 13057**	**Fully vaccinated**<br>**N = 8047**	**Unvaccinated**<br>**N = 4934**	**P-value**
Age: Mean (SD)	20.51 (1.25)	20.52 (1.26)	20.50 (1.24)	0.34[*]
Race/Ethnicity: N (%)				<0.001[**]
...White, non-Hispanic	10808(82.8%)	6533(81.2%)	4217(85.5%)	<0.001[**]
...Black, non-Hispanic	696(5.3%)	469(5.8%)	219(4.4%)	<0.001[**]
...Any race, Hispanic	767(5.9%)	497(6.2%)	263(5.3%)	0.05[**]
...All other, races non-Hispanic	786(6.0%)	548(6.8%)	235(4.8%)	<0.001[**]
Gender: N (%)				<0.001[**]
...Female	6670(51.1%)	4513(56.1%)	2125(43.1%)	<0.001[**]
...Male	6364(48.7%)	3525(43.8%)	2795(56.6%)	<0.001[**]
...Not reported	23(0.2%)	9(0.1%)	14(0.3%)	0.04[**]
Affiliation: N (%)				<0.001[**]
...Residential	1546(11.8%)	1087(13.5%)	448(9.1%)	<0.001[**]
...Non-residential	11511(88.2%)	6960(86.5%)	4486(90.9%)	<0.001[**]
**Condition impacting immune response: N (%)**[♦]	374(2.9%)	215(2.7%)	156(3.2%)	0.12[**]
...Lung disease	78(0.6%)	57(0.7%)	19(0.4%)	0.03[**]
...HIV	2(0.0%)	2(0.0%)	0(0.0%)	0.70[**]
...Cancer	4(0.0%)	1(0.0%)	3(0.1%)	0.31[**]
...Lupus	4(0.0%)	4(0.0%)	0(0.0%)	0.29[**]
...Rheumatoid arthritis	21(0.2%)	11(0.1%)	10(0.2%)	0.49[**]
...Solid organ or bone marrow transplant	8(0.1%)	4(0.0%)	4(0.1%)	0.74[**]
**Any other pre-existing condition: N (%)**[▪]	781(6.0%)	527(6.5%)	247(5.0%)	<0.001[**]
...High blood pressure	92(0.7%)	59(0.7%)	30(0.6%)	0.47[**]
...Heart disease	15(0.1%)	8(0.1%)	7(0.1%)	0.67[**]
...Diabetes	57(0.4%)	41(0.5%)	15(0.3%)	0.11[**]
...Overweight	337(2.6%)	251(3.1%)	85(1.7%)	<0.001[**]
...Kidney disease	12(0.1%)	8(0.1%)	4(0.1%)	0.97[**]
...Cough inefficacy	2(0.0%)	2(0.0%)	0(0.0%)	0.70[**]
...Liver disease	6(0.0%)	5(0.1%)	1(0.0%)	0.51[**]
**Medications**[▲] **N (%)**	407(3.1%)	256(3.2%)	148(3.0%)	0.60[**]
...Steroids	56(0.4%)	44(0.5%)	11(0.2%)	0.009[**]
...Chemotherapy	2(0.0%)	2(0.0%)	0(0.0%)	0.70[**]
...Immunosuppressants	67(0.5%)	45(0.6%)	22(0.4%)	0.45[**]
**Use of tobacco or nicotine products: N (%)**	931(7.1%)	428(5.3%)	500(10.1%)	<0.001[**]
**SARS-CoV-2 Tests Per Person: Mean (SD)**	29.52 (10.95)	31.96 (10.64)	25.58 (10.30)	<0.001[*]
...Fall 2020 Semester	5.09 (3.26)	5.51 (3.33)	4.40 (3.02)	<0.001[*]
...Spring 2021 Semester	10.74 (5.42)	11.53 (5.45)	9.41 (5.11)	<0.001[*]
...Fall 2021 Semester[¶]	13.12 (4.49)	14.08 (3.86)	11.57 (4.97)	<0.001[*]
**Previous SARS-CoV-2 Infection: N (%)**[†]	4320(33.1%)	2531(31.5%)	1762(35.7%)	<0.001[**]
**SARS-CoV-2 Infections During Follow-up: N (%)**[#]	804(6.2%)	256(3.2%)	540(10.9%)	<0.001[**]

♦ Self-reported presence of any condition impacting immune response: HIV, Cancer, Lupus, Rheumatoid Arthritis, Solid organ or bone marrow transplant; sample size may not add to N due to non-selection of specific conditions.

▪ Self-reported presence of any of the following conditions: high blood pressure, heart disease, diabetes, overweight or obesity, kidney disease or dialysis, previous stroke or other neurological condition affecting my ability to cough, liver disease, or lung disease; sample size may not add to N due to non-selection of specific conditions.

▲ Self-reported medication use of any of the following: steroids, chemotherapy, immunosuppressants; sample size may not add to N due to non-selection of specific medications.

¶ During follow-up period (8/8/2021 to 12/4/2021)

† Infection occurring prior to follow-up period (8/7/21)

% is proportion of individuals within each population infected with SARS-CoV-2 during follow-up period

* Based on independent two-sample t-test (2-sided p-value)

** Based on chi-squared test for independence

Table S3: Descriptive characteristics for study sample by vaccine manufacturer between August 8th, 2021 and December 4th, 2021, restricted to individuals with a SARS-CoV-2 testing history prior to November 25th, 2020.

**Characteristic**	**Fully vaccinated N = 8047**	**mRNA-1273 N = 3291**	**BNT162b2 N = 4160**	**Ad26.COV2.S N = 596**	**P-value**
Age: Mean (SD)	20.52 (1.26)	20.65 (1.31)	20.41 (1.21)	20.59 (1.26)	<0.001*
Race/Ethnicity: N (%)					0.02**
...White, non-Hispanic	6533(81.2%)	2665(81.0%)	3353(80.6%)	515(86.4%)	0.002**
...Black, non-Hispanic	469(5.8%)	191(5.8%)	261(6.3%)	17(2.9%)	0.002**
...Any race, Hispanic	497(6.2%)	204(6.2%)	262(6.3%)	31(5.2%)	0.60**
...All other, races non-Hispanic	548(6.8%)	231(7.0%)	284(6.8%)	33(5.5%)	0.43**
Gender: N (%)					<0.001**
...Female	4513(56.1%)	1844(56.0%)	2450(58.9%)	219(36.7%)	<0.001**
...Male	3525(43.8%)	1442(43.8%)	1708(41.1%)	375(62.9%)	<0.001**
...Not reported	9(0.1%)	5(0.2%)	2(0.0%)	2(0.3%)	0.07**
Affiliation: N (%)					<0.001**
...Residential	1087(13.5%)	407(12.4%)	620(14.9%)	60(10.1%)	<0.001**
...Non-residential	6960(86.5%)	2884(87.6%)	3540(85.1%)	536(89.9%)	<0.001**
**Condition impacting immune response: N (%)♦**	215(2.7%)	80(2.4%)	112(2.7%)	23(3.9%)	0.14**
...Lung disease	57(0.7%)	28(0.9%)	27(0.6%)	2(0.3%)	0.38**
...HIV	2(0.0%)	1(0.0%)	0(0.0%)	1(0.2%)	0.07**
...Cancer	1(0.0%)	0(0.0%)	1(0.0%)	0(0.0%)	1.00**
...Lupus	4(0.0%)	2(0.1%)	1(0.0%)	1(0.2%)	0.27**
...Rheumatoid arthritis	11(0.1%)	4(0.1%)	6(0.1%)	1(0.2%)	0.90**
...Solid organ or bone marrow transplant	4(0.0%)	2(0.1%)	2(0.0%)	0(0.0%)	1.00**
**Any other pre-existing condition: N (%)▪**	527(6.5%)	234(7.1%)	253(6.1%)	40(6.7%)	0.20**
...High blood pressure	59(0.7%)	32(1.0%)	21(0.5%)	6(1.0%)	0.04**
...Heart disease	8(0.1%)	4(0.1%)	4(0.1%)	0(0.0%)	0.86**
...Diabetes	41(0.5%)	18(0.5%)	21(0.5%)	2(0.3%)	0.88**
...Overweight	251(3.1%)	118(3.6%)	116(2.8%)	17(2.9%)	0.14**
...Kidney disease	8(0.1%)	3(0.1%)	4(0.1%)	1(0.2%)	0.72**
...Cough inefficacy	2(0.0%)	1(0.0%)	1(0.0%)	0(0.0%)	1.00**
...Liver disease	5(0.1%)	1(0.0%)	4(0.1%)	0(0.0%)	0.59**
**Medications▲ N (%)**	256(3.2%)	97(2.9%)	141(3.4%)	18(3.0%)	0.55**
...Steroids	44(0.5%)	17(0.5%)	27(0.6%)	0(0.0%)	0.10**
...Chemotherapy	2(0.0%)	0(0.0%)	2(0.0%)	0(0.0%)	0.58**
...Immunosuppressants	45(0.6%)	18(0.5%)	24(0.6%)	3(0.5%)	1.00**
**Use of tobacco or nicotine products: N (%)**	428(5.3%)	188(5.7%)	198(4.8%)	42(7.0%)	0.03**
**SARS-CoV-2 Tests Per Person: Mean (SD)**	31.96 (10.64)	32.48 (10.58)	31.91 (10.65)	29.46 (10.55)	<0.001*
...Fall 2020 Semester	5.51 (3.33)	5.53 (3.34)	5.57 (3.32)	5.01 (3.21)	<0.001*
...Spring 2021 Semester	11.53 (5.45)	11.72 (5.37)	11.52 (5.46)	10.61 (5.66)	<0.001*
...Fall 2021 Semester¶	14.08 (3.86)	14.30 (3.79)	14.03 (3.87)	13.20 (4.11)	<0.001*
**Previous SARS-CoV-2 Infection: N (%)†**	2531(31.5%)	889(27.0%)	1421(34.2%)	221(37.1%)	<0.001**
**SARS-CoV-2 Infections During Follow-up: N (%)#**	256(3.2%)	86(2.6%)	139(3.3%)	31(5.2%)	0.004**

♦ Self-reported presence of any condition impacting immune response: HIV, Cancer, Lupus, Rheumatoid Arthritis, Solid organ or bone marrow transplant; sample size may not add to N due to non-selection of specific conditions.

▪ Self-reported presence of any of the following conditions: high blood pressure, heart disease, diabetes, overweight or obesity, kidney disease or dialysis, previous stroke or other neurological condition affecting my ability to cough, liver disease, or lung disease; sample size may not add to N due to non-selection of specific conditions.

▲ Self-reported medication use of any of the following: steroids, chemotherapy, immunosuppressants; sample size may not add to N due to non-selection of specific medications.

¶ During follow-up period (8/8/2021 to 12/4/2021)

† Infection occurring prior to follow-up period (8/7/21)

% is proportion of individuals within each population infected with SARS-CoV-2 during follow-up period

* Based on ANOVA F-test

** Based on chi-square test for independence

Table S4: Estimated protection from vaccination and previous infection against SARS-CoV-2 infection between August 8th, 2021 and December 4th, 2021, restricted to individuals with a SARS-CoV-2 testing history prior to November 25th, 2020.

Vaccine Protection*	# of Individuals	# Positive (%)	Protection: % (95% CI)
Unvaccinated	4934	540 (10.9%)	*Reference*
Fully Vaccinated	8047	256 (3.2%)	70.1% (65.2-74.3)[a]
... mRNA-1273	3291	86 (2.6%)	76.4% (70.3-81.3)[b]
... BNT162b2	4160	139 (3.3%)	68.1% (61.4-73.6)[b]
... Ad26.COV2.S	596	31 (5.2%)	46.6% (23.2-62.8)[b]
**Protection by Vaccination and Previous Infection History**[†]			
**No protection**	3172	465 (14.7%)	*Reference*
**Fully vaccinated**			
...No previous infection	5516	240 (4.4%)	68.2% (62.8-72.9)[c]
...... mRNA-1273	2402	80 (3.3%)	75.5% (68.9-80.7)[d]
...... BNT162b2	2739	129 (4.7%)	66.0% (58.5-72.1)[d]
...... Ad26.COV2.S	375	31 (8.3%)	36.6% (8.8-55.9)[¶d]
...Previous infection	2531	16 (0.6%)	95.6% (92.7-97.3)[c]
...... mRNA-1273	889	6 (0.7%)	95.0% (89.1-97.7)[d]
...... BNT162b2	1421	10 (0.7%)	95.0% (90.7-97.3)[d]
...... Ad26.COV2.S	221	0 (0%)	98.5% (75.6-99.9)[¶d]
**Previous infection only**	1762	75 (4.3%)	74.4% (67.3-80.0)[d]

*Protection is relative to unvaccinated individuals
[†]Protection is relative to individuals with no protection (unvaccinated with no previous SARS-CoV-2 infection)
[¶]Estimates and confidence intervals obtained from Cox regression model with Firth's penalized likelihood method.
[a] Estimated via Model 1.1 in Appendix 1
[b] Estimated via Model 1.2 in Appendix 1
[c] Estimated via Model 1.3 in Appendix 1
[d] Estimated via Model 1.4 in Appendix 1

Table S5: Estimated protection from vaccination and previous infection against SARS-CoV-2 infection between August 8th, 2021 and December 4th, 2021, restricted to individuals with a SARS-CoV-2 testing history prior to November 25th, 2020, and excluding individuals receiving their first vaccination dose prior to March 31st, 2021.

**Vaccine Protection***	**# of Individuals**	**# Positive (%)**	**Protection: % (95% CI)**
Unvaccinated	4934	540 (10.9%)	*Reference*
Fully Vaccinated	7235	224 (3.1%)	70.7% (65.7-75.0)[a]
... mRNA-1273	2997	80 (2.7%)	75.8% (69.3-80.9)[b]
... BNT162b2	3683	115 (3.1%)	70.0% (63.1-75.5)[b]
... Ad26.COV2.S	555	29 (5.2%)	46.2% (21.7-63.0)[b]
**Protection by Vaccination and Previous Infection History**[†]			
**No protection**	3172	465 (14.7%)	*Reference*
**Fully vaccinated**			
...No previous infection	4885	208 (4.3%)	69.1% (63.5-73.8)[c]
...... mRNA-1273	2158	74 (3.4%)	74.9% (67.8-80.4)[d]
...... BNT162b2	2383	105 (4.4%)	68.3% (60.7-74.5)[d]
...... Ad26.COV2.S	344	29 (8.4%)	35.7% (6.6-55.8)[¶d]
...Previous infection	2350	16 (0.7%)	95.2% (92.1-97.1)[c]
...... mRNA-1273	839	6 (0.7%)	94.7% (88.4-97.5)[d]
...... BNT162b2	1300	10 (0.8%)	94.5% (89.8-97.0)[d]
...... Ad26.COV2.S	211	0 (0%)	98.4% (74.3-99.9)[¶d]
**Previous infection only**	1762	75 (4.3%)	74.3% (67.2-79.9)[d]

*Protection is relative to unvaccinated individuals

[†]Protection is relative to individuals with no protection (unvaccinated with no previous SARS-CoV-2 infection)

[¶]Estimates and confidence intervals obtained from Cox regression model with Firth's penalized likelihood method.

[a] Estimated via Model 1.1 in Appendix 1

[b] Estimated via Model 1.2 in Appendix 1

[c] Estimated via Model 1.3 in Appendix 1

[d] Estimated via Model 1.4 in Appendix 1

Table S6: Descriptive characteristics for study sample between December 28th, 2020 and December 4th, 2021, restricted to individuals with a SARS-CoV-2 testing history prior to November 25th, 2020.

Characteristic	Total N = 13191	Fully vaccinated N = 7331	Unvaccinated N = 5768	P-value
Age: Mean (SD)	20.46 (1.32)	20.54 (1.28)	20.34 (1.35)	<0.001*
Race/Ethnicity: N (%)				<0.001**
...White, non-Hispanic	10895(82.6%)	5931(80.9%)	4900(85.0%)	<0.001**
...Black, non-Hispanic	704(5.3%)	436(5.9%)	259(4.5%)	<0.001**
...Any race, Hispanic	782(5.9%)	455(6.2%)	316(5.5%)	0.09**
...All other, races non-Hispanic	810(6.1%)	509(6.9%)	293(5.1%)	<0.001**
Gender: N (%)				<0.001**
...Female	6724(51.0%)	4119(56.2%)	2569(44.5%)	<0.001**
...Male	6441(48.8%)	3203(43.7%)	3182(55.2%)	<0.001**
...Not reported	26(0.2%)	9(0.1%)	17(0.3%)	0.05**
Affiliation: N (%)				<0.001**
...Residential	1546(11.7%)	973(13.3%)	561(9.7%)	<0.001**
...Non-residential	11645(88.3%)	6358(86.7%)	5207(90.3%)	<0.001**
**Condition impacting immune response: N (%)♦**	376(2.9%)	192(2.6%)	181(3.1%)	0.09**
...Lung disease	79(0.6%)	49(0.7%)	27(0.5%)	0.17**
...HIV	2(0.0%)	2(0.0%)	0(0.0%)	0.59**
...Cancer	4(0.0%)	1(0.0%)	3(0.1%)	0.46**
...Lupus	4(0.0%)	3(0.0%)	1(0.0%)	0.79**
...Rheumatoid arthritis	22(0.2%)	8(0.1%)	14(0.2%)	0.10**
...Solid organ or bone marrow transplant	8(0.1%)	3(0.0%)	5(0.1%)	0.49**
**Any other pre-existing condition: N (%)▪**	790(6.0%)	476(6.5%)	303(5.3%)	0.003**
...High blood pressure	93(0.7%)	52(0.7%)	39(0.7%)	0.90**
...Heart disease	15(0.1%)	8(0.1%)	7(0.1%)	1.00**
...Diabetes	58(0.4%)	36(0.5%)	20(0.3%)	0.26**
...Overweight	342(2.6%)	230(3.1%)	109(1.9%)	<0.001**
...Kidney disease	12(0.1%)	7(0.1%)	5(0.1%)	1.00**
...Cough inefficacy	2(0.0%)	2(0.0%)	0(0.0%)	0.59**
...Liver disease	6(0.0%)	4(0.1%)	2(0.0%)	0.91**
**Medications▲ N (%)**	412(3.1%)	234(3.2%)	175(3.0%)	0.64**
...Steroids	58(0.4%)	42(0.6%)	15(0.3%)	0.01**
...Chemotherapy	2(0.0%)	2(0.0%)	0(0.0%)	0.59**
...Immunosuppressants	67(0.5%)	42(0.6%)	25(0.4%)	0.32**
**Use of tobacco or nicotine products: N (%)**	946(7.2%)	383(5.2%)	563(9.8%)	<0.001**
**SARS-CoV-2 Tests Per Person: Mean (SD)**	29.35 (11.04)	32.37 (10.76)	25.56 (10.19)	<0.001*
...Fall 2020 Semester	5.07 (3.25)	5.43 (3.32)	4.62 (3.11)	<0.001*
...Spring 2021 Semester	10.71 (5.42)	12.16 (5.14)	8.82 (5.21)	<0.001*
...Fall 2021 Semester¶	24.16 (9.19)	26.80 (8.78)	20.84 (8.60)	<0.001*
**Previous SARS-CoV-2 Infection: N (%)†**	3090(23.4%)	1779(24.3%)	1288(22.3%)	0.01**
**SARS-CoV-2 Infections During Follow-up: N (%)#**	2110(16.0%)	267(3.6%)	1826(31.7%)	<0.001**

♦ Self-reported presence of any condition impacting immune response: HIV, Cancer, Lupus, Rheumatoid Arthritis, Solid organ or bone marrow transplant; sample size may not add to N due to non-selection of specific conditions.

▪ Self-reported presence of any of the following conditions: high blood pressure, heart disease, diabetes, overweight or obesity, kidney disease or dialysis, previous stroke or other neurological condition affecting my ability to cough, liver disease, or lung disease; sample size may not add to N due to non-selection of specific conditions.

▲ Self-reported medication use of any of the following: steroids, chemotherapy, immunosuppressants; sample size may not add to N due to non-selection of specific medications.

¶ During follow-up period (12/28/2020 to 12/4/2021)

† Infection occurring prior to follow-up period (12/27/20)

% is proportion of individuals within each population infected with SARS-CoV-2 during follow-up period

* Based on independent two-sample t-test (2-sided p-value)

** Based on chi-squared test for independence

Table S7: Descriptive characteristics for study sample by vaccine manufacturer between December 28th, 2020 and December 4th, 2021, restricted to individuals with a SARS-CoV-2 testing history prior to November 25th, 2020.

**Characteristic**	**Fully vaccinated N = 7331**	**mRNA-1273 N = 3029**	**BNT162b2 N = 3762**	**Ad26.COV2.S N = 540**	**P-value**
Age: Mean (SD)	20.54 (1.28)	20.67 (1.33)	20.43 (1.23)	20.56 (1.26)	<0.001*
Race/Ethnicity: N (%)					0.007**
...White, non-Hispanic	5931(80.9%)	2444(80.7%)	3017(80.2%)	470(87.0%)	<0.001**
...Black, non-Hispanic	436(5.9%)	178(5.9%)	243(6.5%)	15(2.8%)	0.001**
...Any race, Hispanic	455(6.2%)	190(6.3%)	238(6.3%)	27(5.0%)	0.51**
...All other, races non-Hispanic	509(6.9%)	217(7.2%)	264(7.0%)	28(5.2%)	0.24**
Gender: N (%)					<0.001**
...Female	4119(56.2%)	1699(56.1%)	2217(58.9%)	203(37.6%)	<0.001**
...Male	3203(43.7%)	1324(43.7%)	1543(41.0%)	336(62.2%)	<0.001**
...Not reported	9(0.1%)	6(0.2%)	2(0.1%)	1(0.2%)	0.15**
Affiliation: N (%)					<0.001**
...Residential	973(13.3%)	368(12.1%)	550(14.6%)	55(10.2%)	0.001**
...Non-residential	6358(86.7%)	2661(87.9%)	3212(85.4%)	485(89.8%)	0.001**
**Condition impacting immune response: N (%)♦**	192(2.6%)	72(2.4%)	98(2.6%)	22(4.1%)	0.08**
...Lung disease	49(0.7%)	24(0.8%)	23(0.6%)	2(0.4%)	0.52**
...HIV	2(0.0%)	1(0.0%)	0(0.0%)	1(0.2%)	0.07**
...Cancer	1(0.0%)	0(0.0%)	1(0.0%)	0(0.0%)	1.00**
...Lupus	3(0.0%)	1(0.0%)	1(0.0%)	1(0.2%)	0.28**
...Rheumatoid arthritis	8(0.1%)	2(0.1%)	6(0.2%)	0(0.0%)	0.53**
...Solid organ or bone marrow transplant	3(0.0%)	2(0.1%)	1(0.0%)	0(0.0%)	0.67**
**Any other pre-existing condition: N (%)▪**	476(6.5%)	214(7.1%)	225(6.0%)	37(6.9%)	0.18**
...High blood pressure	52(0.7%)	31(1.0%)	16(0.4%)	5(0.9%)	0.008**
...Heart disease	8(0.1%)	4(0.1%)	4(0.1%)	0(0.0%)	1.00**
...Diabetes	36(0.5%)	17(0.6%)	17(0.5%)	2(0.4%)	0.81**
...Overweight	230(3.1%)	108(3.6%)	107(2.8%)	15(2.8%)	0.22**
...Kidney disease	7(0.1%)	3(0.1%)	3(0.1%)	1(0.2%)	0.56**
...Cough inefficacy	2(0.0%)	1(0.0%)	1(0.0%)	0(0.0%)	1.00**
...Liver disease	4(0.1%)	1(0.0%)	3(0.1%)	0(0.0%)	0.73**
**Medications▲ N (%)**	234(3.2%)	91(3.0%)	126(3.3%)	17(3.1%)	0.73**
...Steroids	42(0.6%)	17(0.6%)	25(0.7%)	0(0.0%)	0.14**
...Chemotherapy	2(0.0%)	0(0.0%)	2(0.1%)	0(0.0%)	0.58**
...Immunosuppressants	42(0.6%)	17(0.6%)	23(0.6%)	2(0.4%)	0.89**
**Use of tobacco or nicotine products: N (%)**	383(5.2%)	168(5.5%)	180(4.8%)	35(6.5%)	0.14**
**SARS-CoV-2 Tests Per Person: Mean (SD)**	32.37 (10.76)	32.89 (10.67)	32.35 (10.83)	29.66 (10.43)	<0.001*
...Fall 2020 Semester	5.43 (3.32)	5.47 (3.33)	5.47 (3.32)	4.91 (3.15)	<0.001*
...Spring 2021 Semester	12.16 (5.14)	12.30 (5.09)	12.20 (5.13)	11.14 (5.35)	<0.001*
...Fall 2021 Semester¶	26.80 (8.78)	27.27 (8.64)	26.74 (8.86)	24.62 (8.68)	<0.001*
**Previous SARS-CoV-2 Infection: N (%)†**	1779(24.3%)	610(20.1%)	1008(26.8%)	161(29.8%)	<0.001**
**SARS-CoV-2 Infections During Follow-up: N (%)#**	267(3.6%)	93(3.1%)	141(3.7%)	33(6.1%)	0.003**

♦ Self-reported presence of any condition impacting immune response: HIV, Cancer, Lupus, Rheumatoid Arthritis, Solid organ or bone marrow transplant; sample size may not add to N due to non-selection of specific conditions.

▪ Self-reported presence of any of the following conditions: high blood pressure, heart disease, diabetes, overweight or obesity, kidney disease or dialysis, previous stroke or other neurological condition affecting my ability to cough, liver disease, or lung disease; sample size may not add to N due to non-selection of specific conditions.

▲ Self-reported medication use of any of the following: steroids, chemotherapy, immunosuppressants; sample size may not add to N due to non-selection of specific medications.

¶ During follow-up period (12/28/2020 to 12/4/2021)

† Infection occurring prior to follow-up period (12/27/20)

\# % is proportion of individuals within each population infected with SARS-CoV-2 during follow-up period

* Based on ANOVA F-test

** Based on chi-square test for independence

Table S8: Estimated protection from vaccination and previous infection against SARS-CoV-2 infection between December 28th, 2020 and December 4th, 2021, restricted to individuals with a SARS-CoV-2 testing history prior to November 25th, 2020.

**Vaccine Protection***	**# of Individuals**	**# Positive (%)**	**Protection: % (95% CI)**
Unvaccinated	5768	1826 (31.7%)	*Reference*
Fully Vaccinated	7331	267 (3.6%)	69.8% (65.0-73.9)[a]
... mRNA-1273	3029	93 (3.1%)	74.6% (68.4-79.6)[b]
... BNT162b2	3762	141 (3.7%)	69.1% (62.8-74.3)[b]
... Ad26.COV2.S	540	33 (6.1%)	46.6% (24.2-62.4)[b]
**Protection by Vaccination and Previous Infection History†**			
**No protection**	4480	1698 (37.9%)	*Reference*
**Fully vaccinated**			
...No previous infection	5552	256 (4.6%)	68.8% (63.8-73.1)[c]
...... mRNA-1273	2419	88 (3.6%)	74.4% (67.9-79.6)[d]
...... BNT162b2	2754	135 (4.9%)	67.9% (61.2-73.4)[d]
...... Ad26.COV2.S	379	33 (8.7%)	40.2% (15.1-57.8)[¶d]
...Previous infection	1779	11 (0.6%)	96.3% (93.4-98.0)[c]
...... mRNA-1273	610	5 (0.8%)	94.7% (87.8-97.7)[d]
...... BNT162b2	1008	6 (0.6%)	96.3% (92.1-98.3)[d]
...... Ad26.COV2.S	161	0 (0%)	98.4% (73.8-99.9)[d]
**Previous infection only**	1288	128 (9.9%)	80.9% (77.1-84.0)[¶d]

*Protection is relative to unvaccinated individuals

†Protection is relative to individuals with no protection (unvaccinated with no previous SARS-CoV-2 infection)

¶Estimates and confidence intervals obtained from Cox regression model with Firth's penalized likelihood method.

[a] Estimated via Model 1.1 in Appendix 1

[b] Estimated via Model 1.2 in Appendix 1

[c] Estimated via Model 1.3 in Appendix 1

[d] Estimated via Model 1.4 in Appendix 1

Table S9: Estimated protection from vaccination and previous infection against SARS-CoV-2 infection between December 28th, 2020 and December 4th, 2021, restricted to individuals with a SARS-CoV-2 testing history prior to November 25th, 2020, and excluding individuals receiving their first vaccination dose prior to March 31st, 2021.

**Vaccine Protection***	**# of Individuals**	**# Positive (%)**	**Protection: % (95% CI)**
Unvaccinated	5724	1789 (31.3%)	*Reference*
Fully Vaccinated	6548	230 (3.5%)	70.7% (65.8-74.9)[a]
... mRNA-1273	2747	84 (3.1%)	74.6% (68.0-79.8)[b]
... BNT162b2	3302	116 (3.5%)	70.9% (64.5-76.2)[b]
... Ad26.COV2.S	499	30 (6%)	47.0% (23.5-63.3)[b]
**Protection by Vaccination and Previous Infection History†**			
**No protection**	4438	1663 (37.5%)	*Reference*
**Fully vaccinated**			
...No previous infection	4906	219 (4.5%)	69.9% (64.7-74.3)[c]
...... mRNA-1273	2170	79 (3.6%)	74.4% (67.6-79.8)[d]
...... BNT162b2	2391	110 (4.6%)	70.0% (63.1-75.6)[d]
...... Ad26.COV2.S	345	30 (8.7%)	40.4% (14.0-58.6)[¶d]
...Previous infection	1642	11 (0.7%)	96.0% (92.8-97.8)[c]
...... mRNA-1273	577	5 (0.9%)	94.4% (87.0-97.6)[d]
...... BNT162b2	911	6 (0.7%)	96.0% (91.3-98.2)[d]
...... Ad26.COV2.S	154	0 (0%)	98.3% (72.1-99.9)[¶d]
**Previous infection only**	1286	126 (9.8%)	81.0% (77.2-84.1)[d]

*Protection is relative to unvaccinated individuals

†Protection is relative to individuals with no protection (unvaccinated with no previous SARS-CoV-2 infection)

¶Estimates and confidence intervals obtained from Cox regression model with Firth's penalized likelihood method.

[a] Estimated via Model 1.1 in Appendix 1

[b] Estimated via Model 1.2 in Appendix 1

[c] Estimated via Model 1.3 in Appendix 1

[d] Estimated via Model 1.4 in Appendix 1

Table S10: Estimated hazard ratio (HR) for increase in monthly risk of infection.

	**Main Analysis (8/8/21-12/4/21)**	**Sensitivity Analysis 1[♦] (8/8/21-12/4/21)**	**Sensitivity Analysis 2[▪] (8/8/21-12/4/21)**	**Sensitivity Analysis 1[♦] (12/28/20-12/4/21)**	**Sensitivity Analysis 2[▪] (12/28/20-12/4/21)**
**Status**		**HR (95% CI)**	**HR (95% CI)**	**HR (95% CI)**	**HR (95% CI)**
All Vaccines[*†a]	1.15 (1.08-1.23)	1.17 (1.07-1.27)	1.18 (1.05-1.32)	1.22 (1.13-1.33)	1.23 (1.10-1.38)
... mRNA-1273[b]	1.25 (1.10-1.42)	1.24 (1.05-1.46)	1.30 (1.07-1.57)	1.31 (1.12-1.53)	1.36 (1.13-1.63)
... BNT162b2[b]	1.17 (1.09-1.26)	1.20 (1.08-1.34)	1.24 (1.07-1.43)	1.28 (1.15-1.42)	1.28 (1.11-1.48)
... Ad26.COV2.S[b]	0.94 (0.81-1.09)	0.87 (0.69-1.09)	0.87 (0.68-1.11)	0.93 (0.76-1.14)	0.91 (0.72-1.16)
Previous Infection[#¶b]	0.94 (0.87-1.02)	0.96 (0.88-1.05)	0.97 (0.89-1.05)	0.92 (0.85-0.98)	0.92 (0.86-0.99)

[♦] All students (18-24 years of age) undergoing surveillance testing during follow-up, restricted to individuals with a SARS-CoV-2 testing history prior to November 25th, 2020.

[▪] All students (18-24 years of age) undergoing surveillance testing during follow-up, restricted to individuals with a SARS-CoV-2 testing history prior to November 25th, 2020, and excluding individuals receiving their first vaccination dose prior to March 31st, 2021.

*Protection is relative to unvaccinated individuals

[†] Monthly risk estimated by hazard ratio (HR) for months since vaccination.

[#] Protection is relative to individuals with no previous SARS-CoV-2 infection

[¶] Monthly risk estimated by hazard ratio (HR) for months since previous infection.

[a] Estimated via Model 2.1 in Appendix 2

[b] Estimated via Model 2.2 in Appendix 2

Table S11: Comparison of estimated protection from vaccination and previous infection against SARS-CoV-2 infection between August 8, 2021 and December 4, 2021 (Main Analysis Sample). Partial vaccination is defined as 7 days after the first dose of mRNA-1273 or BNT162b2. Full vaccination is defined as 7 days after the first dose of Ad26.COV2.S or 7 days after the second dose of mRNA-1273 or BNT162b2.

	**Main analysis (8/8/2021-12/4/2021)**	**Sensitivity analysis 3[♦] (8/8/2021-12/4/2021)**
**Vaccine Protection***	**Protection: % (95% CI)**	**Protection: % (95% CI)**
Unvaccinated	*Reference*	*Reference*
Fully Vaccinated	67.4% (63.7-70.7)[a]	67.9% (64.3-71.2)[a]
... mRNA-1273	75.4% (70.5-79.5)[b]	75.8% (70.9-79.8)[b]
... BNT162b2	65.7% (61.1-69.8)[b]	66.3% (61.8-70.3)[b]
... Ad26.COV2.S	42.8% (26.1-55.8)[b]	44.0% (27.6-56.7)[b]
**Protection by Vaccination and Previous Infection History[†]**		
**No protection**	*Reference*	*Reference*
**Fully vaccinated**		
...No previous infection	66.2% (62.3-69.7)[c]	66.8% (62.9-70.2)[c]
...... mRNA-1273	74.9% (69.7-79.2)[d]	75.2% (70.1-79.5)[d]
...... BNT162b2	64.5% (59.6-68.8)[d]	65.2% (60.3-69.4)[d]
...... Ad26.COV2.S	37.2% (18.9-51.4)[¶d]	38.4% (20.4-52.4)[d]
...Previous infection	95.0% (92.1-96.9)[c]	95.1% (92.3-96.9)[c]
...... mRNA-1273	94.3% (88.3-97.2)[d]	94.4% (88.5-97.3)[d]
...... BNT162b2	94.5% (90.2-97.0)[d]	94.7% (90.4-97.0)[d]
...... Ad26.COV2.S	98.5% (75.7-99.9)[¶d]	98.5% (76.2-99.9)[d]
**Previous infection only**	72.9% (66.1-78.4)[d]	72.6% (65.7-78.1)[d]

♦ All students (18-24 years of age) undergoing surveillance testing during follow-up. Partial vaccination is defined as 7 days after the first dose of mRNA-1273 or BNT162b2. Full vaccination is defined as 7 days after the first dose of Ad26.COV2.S or 7 days after the second dose of mRNA-1273 or BNT162b2.

*Protection is relative to unvaccinated individuals

†Protection is relative to individuals with no protection (unvaccinated with no previous SARS-CoV-2 infection)

¶Estimates and confidence intervals obtained from Cox regression model with Firth's penalized likelihood method.

a Estimated via Model 1.1 in Appendix 1

b Estimated via Model 1.2 in Appendix 1

c Estimated via Model 1.3 in Appendix 1

d Estimated via Model 1.4 in Appendix 1

Table S12: Comparison of estimated hazard ratio (HR) for increase in monthly risk of infection (Main Analysis Sample).

	**Main Analysis (8/8/21-12/4/21)**	**Sensitivity Analysis 3[♦] (8/8/21-12/4/21)**
**Status**	**HR (95% CI)**	**HR (95% CI)**
All Vaccines*[†a]	1.15 (1.08-1.23)	1.16 (1.09-1.24)
... mRNA-1273[b]	1.25 (1.10-1.42)	1.24 (1.09-1.40)
... BNT162b2[b]	1.17 (1.09-1.26)	1.19 (1.10-1.28)
... Ad26.COV2.S[b]	0.94 (0.81-1.09)	0.96 (0.83-1.11)
Previous Infection[#¶b]	0.94 (0.87-1.02)	0.94 (0.87-1.02)

[♦]All students (18-24 years of age) undergoing surveillance testing during follow-up. Partial vaccination is defined as 7 days after the first dose of mRNA-1273 or BNT162b2. Full vaccination is defined as 7 days after the first dose of Ad26.COV2.S or 7 days after the second dose of mRNA-1273 or BNT162b2.

*Protection is relative to unvaccinated individuals

[†]Monthly risk estimated by hazard ratio (HR) for months since vaccination.

[#] Protection is relative to individuals with no previous SARS-CoV-2 infection

[¶] Monthly risk estimated by hazard ratio (HR) for months since previous infection.

[a] Estimated via Model 2.1 in Appendix 2

[b] Estimated via Model 2.2 in Appendix 2

Table S13: Comparison of protection and hazard ratio for time-unadjusted and time-adjusted models (Main Analysis Sample).

	**Main analysis**	**Time-adjusted**
**Unvaccinated**	*Reference*	*Reference*
**Fully vaccinated***	67.4% (63.7-70.7) [a]	67.2% (63.5-70.5) [e]
…mRNA-1273	75.4% (70.5-79.5) [b]	75.3% (70.3-79.4) [f]
…BNT162b2	65.7% (61.1-69.8) [b]	65.5% (60.8-69.6) [f]
…Ad26.COV2.S	42.8% (26.1-55.8) [b]	42.5% (25.7-55.5) [f]
**Protection from previous infection only¶**	72.9% (66.1-78.4) [d]	74.3% (67.4-79.7) [h]
**HR of vaccination w/ previous infection against vaccination w/out previous infection**		
**Overall**	0.15 (0.09-0.23) [c]	0.15 (0.09-0.24) [g]
…mRNA-1273	0.23 (0.11-0.47) [d]	0.23 (0.11-0.48) [h]
…BNT162b2	0.15 (0.09-0.28) [d]	0.16 (0.09-0.28) [h]
…Ad26.COV2.S	0.02 (0.00-0.39) [d]	0.02 (0.00-0.41) [h]

*Protection is relative to unvaccinated individuals
†Protection is relative to individuals without previous SARS-CoV-2 infections.
¶ Protection is relative to individuals with no protection (unvaccinated with no previous SARS-CoV-2 infection)
[a] Estimated via Model 1.1 in Appendix 1
[b] Estimated via Model 1.2 in Appendix 1
[c] Estimated via Model 1.3 in Appendix 1
[d] Estimated via Model 1.4 in Appendix 1
[e] Estimated via Model 1.5 in Appendix 1
[f] Estimated via Model 1.6 in Appendix 1
[g] Estimated via Model 1.7 in Appendix 1
[h] Estimated via Model 1.8 in Appendix 1

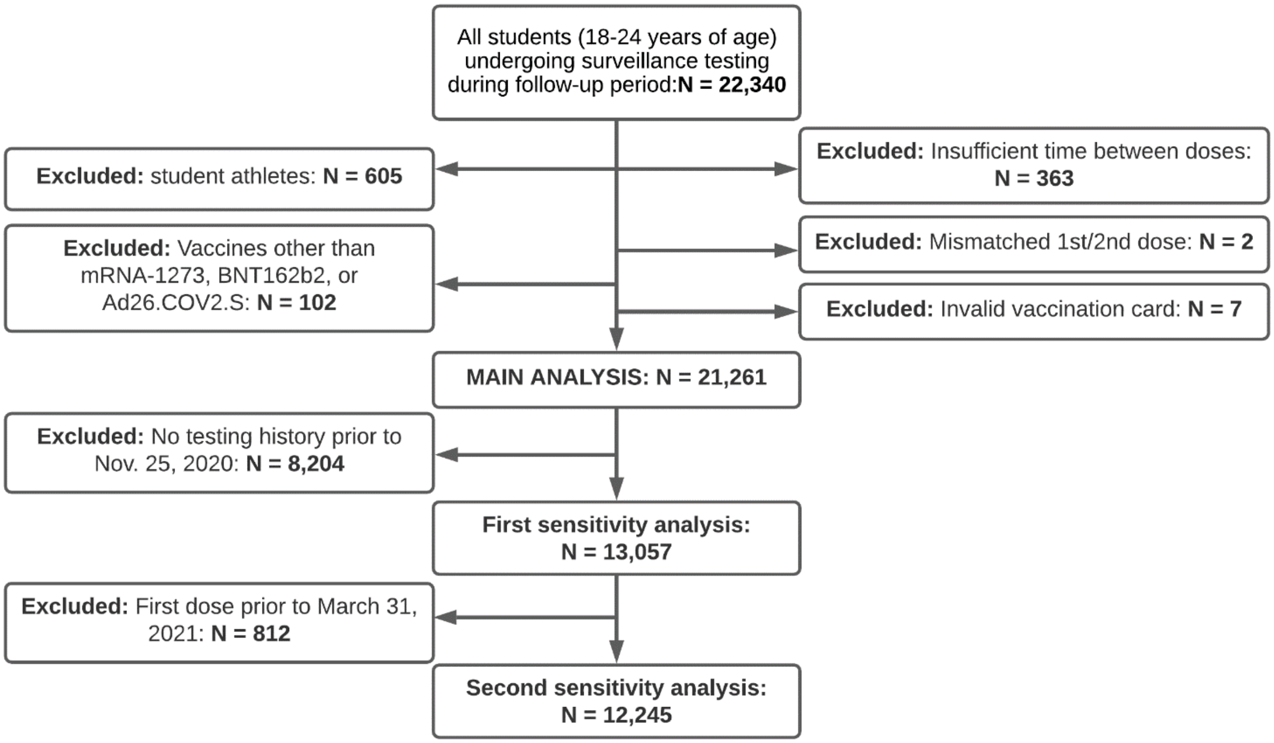

Figure S1: Flow chart of the study samples and exclusion criteria in the main analysis and sensitivity analyses during follow-up between August 8th, 2021 and December 4th, 2021.

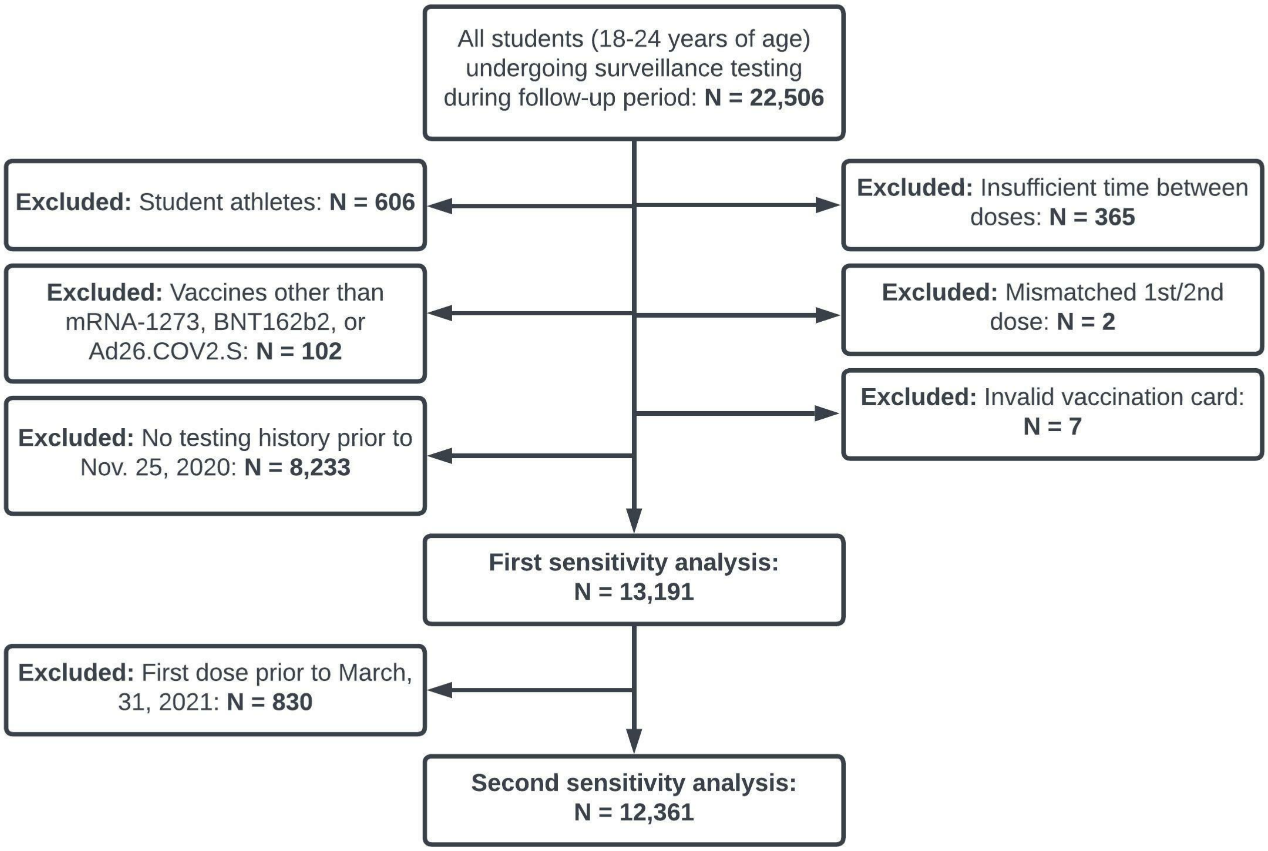

Figure S2: Flow chart of the study samples and exclusion criteria in the sensitivity analyses during follow-up between December 28th, 2020 and December 4th, 2021.

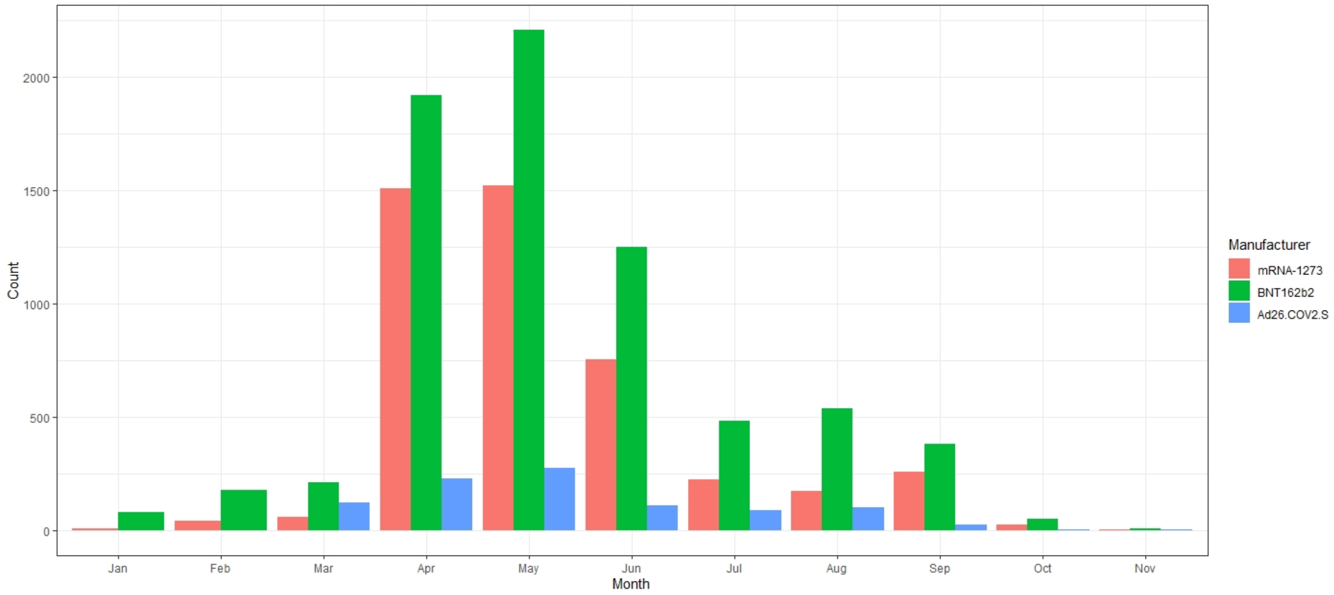

Figure S3: Number of fully vaccinated individuals since January 1st, 2021 in the main analysis sample.
