# Supplementary material for: Effectiveness and protection duration of Covid-19 vaccines and previous infection against any SARS-CoV-2 infection in young adults
Source: Nat Commun. 2022 Jul 8;13:3946. doi: 10.1038/s41467-022-31469-z (PMC9263799; doi:10.1038/s41467-022-31469-z)
Supplement: Supplementary file 3 — Reporting Summary [file 41467_2022_31469_MOESM3_ESM.pdf]

nature portfolio

Corresponding author(s): Lior Rennert

Last updated by author(s): Jan 10, 2022

Reporting Summary

Nature Portfolio wishes to improve the reproducibility of the work that we publish. This form provides structure for consistency and transparency in reporting. For further information on Nature Portfolio policies, see our Editorial Policies and the Editorial Policy Checklist.

Statistics

For all statistical analyses, confirm that the following items are present in the figure legend, table legend, main text, or Methods section.

n/a	Confirmed	
☐	☒	The exact sample size (*n*) for each experimental group/condition, given as a discrete number and unit of measurement
☐	☒	A statement on whether measurements were taken from distinct samples or whether the same sample was measured repeatedly
☐	☒	The statistical test(s) used AND whether they are one- or two-sided<br>*Only common tests should be described solely by name; describe more complex techniques in the Methods section.*
☐	☒	A description of all covariates tested
☐	☒	A description of any assumptions or corrections, such as tests of normality and adjustment for multiple comparisons
☐	☒	A full description of the statistical parameters including central tendency (e.g. means) or other basic estimates (e.g. regression coefficient) AND variation (e.g. standard deviation) or associated estimates of uncertainty (e.g. confidence intervals)
☒	☐	For null hypothesis testing, the test statistic (e.g. F, t, r) with confidence intervals, effect sizes, degrees of freedom and P value noted<br>*Give P values as exact values whenever suitable.*
☒	☐	For Bayesian analysis, information on the choice of priors and Markov chain Monte Carlo settings
☒	☐	For hierarchical and complex designs, identification of the appropriate level for tests and full reporting of outcomes
☒	☐	Estimates of effect sizes (e.g. Cohen's *d*, Pearson's *r*), indicating how they were calculated

Our web collection on statistics for biologists contains articles on many of the points above.

Software and code

Policy information about availability of computer code

Data collection	The methods for data collection are described in the Methods Section of the manuscript.
Data analysis	All analyses were conducted using R version 4.0.5. R packages used in the analyses include tidyverse (v 1.3.1), lubridate (v 1.8.0), survival (v 3.2-13), coxphf (v 1.13.1). R code that runs a simulated data set in a Demo to produce analyses similar to those presented in the Results section of the main manuscript can be found at https://github.com/ZichenMa-USC/Covid19NatureComm

For manuscripts utilizing custom algorithms or software that are central to the research but not yet described in published literature, software must be made available to editors and reviewers. We strongly encourage code deposition in a community repository (e.g. GitHub). See the Nature Portfolio guidelines for submitting code & software for further information.

Data

Policy information about availability of data

All manuscripts must include a data availability statement. This statement should provide the following information, where applicable:

- Accession codes, unique identifiers, or web links for publicly available datasets
- A description of any restrictions on data availability
- For clinical datasets or third party data, please ensure that the statement adheres to our policy

Data collected for this study has not received institutional approval to be shared at this time.

Field-specific reporting

Please select the one below that is the best fit for your research. If you are not sure, read the appropriate sections before making your selection.

- [x] Life sciences
- [ ] Behavioural & social sciences
- [ ] Ecological, evolutionary & environmental sciences

For a reference copy of the document with all sections, see nature.com/documents/nr-reporting-summary-flat.pdf

Life sciences study design

All studies must disclose on these points even when the disclosure is negative.

Sample size	This retrospective study is based on a large population of university students. The sample size was determined by the number of students who participated in mandatory surveillance testing (with some exclusion criteria applied to reduce selection bias; this is described in the manuscript). The final study sample consists of 21,261 students undergoing mandatory SARS-CoV-2 surveillance testing during the 18-week follow-up period.
Data exclusions	Student athletes were excluded from this study (N = 605), since they represent a higher-risk population and undergo more stringent testing protocols. Also excluded were individuals receiving a vaccine dose without emergency use authorization (EUA) from the U.S. Food and Drug Administration (N = 102), individuals with under 21 days between first and second dose of BNT162b2 or under 28 days between first and second dose of mRNA-1273 (N = 363), individuals who received two doses from different manufacturers (N = 2), and individuals with invalid vaccination cards (N = 7).
Replication	This is a retrospective cohort study, and therefore exact reproducibility of the experimental findings of this study are not possible. Studies in other populations have produced similar results to those presented in this paper.
Randomization	This is a retrospective cohort study and the investigators had no control over the participants choice to receive the intervention (i.e., Covid-19 vaccination). Therefore randomization is not applicable to this study.
Blinding	This is a retrospective cohort study and therefore blinding is not applicable.

Reporting for specific materials, systems and methods

We require information from authors about some types of materials, experimental systems and methods used in many studies. Here, indicate whether each material, system or method listed is relevant to your study. If you are not sure if a list item applies to your research, read the appropriate section before selecting a response.

Materials & experimental systems

n/a	Involved in the study	
x		Antibodies
x		Eukaryotic cell lines
x		Palaeontology and archaeology
x		Animals and other organisms
	x	Human research participants
x		Clinical data
x		Dual use research of concern

Methods

n/a	Involved in the study	
x		ChIP-seq
x		Flow cytometry
x		MRI-based neuroimaging

Human research participants

Policy information about studies involving human research participants

Population characteristics	Mean age of the sample was 20.05 (SD=1.55) years, 51.0% identified as female, 48.7% as Male, and 0.3% did not report. The majority of the population was White non-Hispanic (79.6%), 6.0% were Black non-Hispanic, 6.6% were any race Hispanic, and 7.8% were other race non-Hispanic. A total of 1.9% of the sample had a condition impacting immune response, 5.3% had some pre-existing condition, 2.3% were on medications (steroids, chemotherapy, or immunosuppressants), and 5.9% reported use of tobacco or nicotine products. Fully vaccinated individuals compromised of 60.1% of the sample; 21.5% had a record SARS-CoV-2 infection prior to follow-up. Average number of SARS-CoV-2 tests per person prior to the follow-up period was 23.94 (SD=3.27). A total of 7.5% of the population was infected during the follow-up period.
Recruitment	There was no recruitment of participants for this retrospective cohort studies. Participants included all university students participating in Clemson's surveillance testing program. No consent was needed for this study. Students consented to being tested and receiving the Covid-19 vaccine. De-identified data was used for all analyses.
Ethics oversight	Ethical review for this study was obtained by the Institutional Review Board of Clemson University.

Note that full information on the approval of the study protocol must also be provided in the manuscript.
