# Supplementary material for: Effectiveness and protection duration of Covid-19 vaccines and previous infection against any SARS-CoV-2 infection in young adults
Source: Nat Commun. 2022 Jul 8;13:3946. doi: 10.1038/s41467-022-31469-z (PMC9263799; doi:10.1038/s41467-022-31469-z)

0. Preamble

This document includes detailed information on how to install the statistical software R (version 4.1.2) on Windows, install the “tidyverse” R package, and run a simulated data set in a Demo to produce analyses similar to those presented in the Results section of the manuscript. Installation time and running time in this document are estimated using a laptop computer. Using a desktop computer will likely be faster. The code (and corresponding data sets) to generate data Figures 1 and 2 in the main text, and Supplementary Figure 3 in the Supplementary Information, is also included.

1. System requirements

The Demo is based on the software R. The code was written and tested on a Windows 10 operating system for R versions 4.0.5 and 4.1.2 (latest).

2. Installation guide

2.1 Install R (version 4.1.2) on Windows

(1) Go to "https://cran.r-project.org/". Click on "Download R for Windows"

- "install R for the first time"
- "Download R 4.1.2 for Windows"
- Click on “Save File” to download the installer “R-4.1.2-win.exe”.

- Run “R-4.1.2-win.exe” after download is finished. Follow the instructions to complete the installation process (typical timeframe: < 5 minutes).

(2) If the installation is successful, find the “R” folder from the Start Menu and click on “R x64 4.1.2”

And this window will show up.


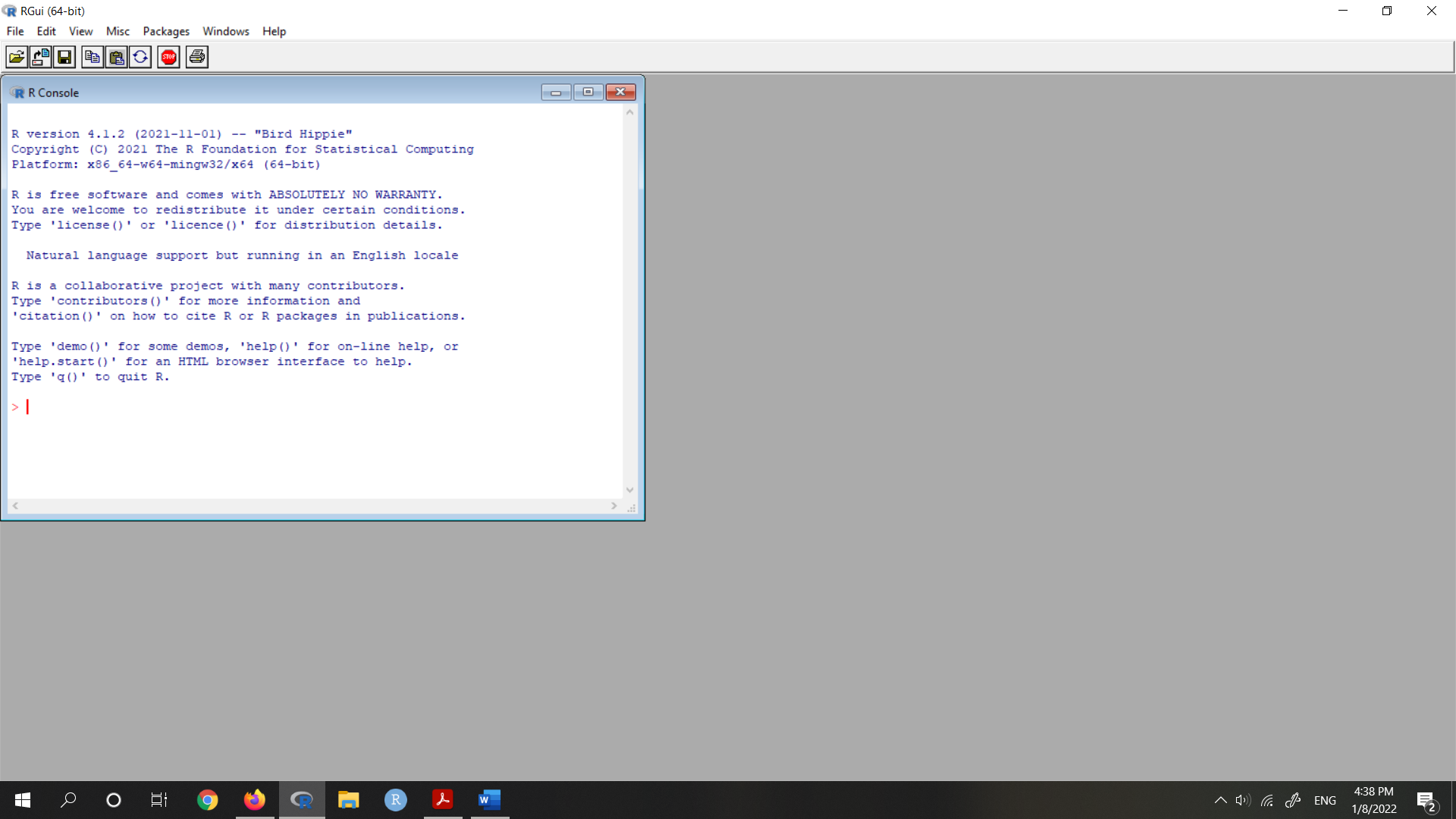


2.2. Install R package “tidyverse”.

(1) Type “install.packages(“tidyverse”)” in R Console. Press Enter. Click “OK” in the “Secure CRAN mirrors” window. If this is your first time installing an R package, you may be asked if you want to create a personal R library. Click “Yes” if that window pops up. Wait until the package is successfully installed--tidyverse is a relatively big package. And several dependent packages will be installed (automatically) in this process (~ 5 minutes of installation time).


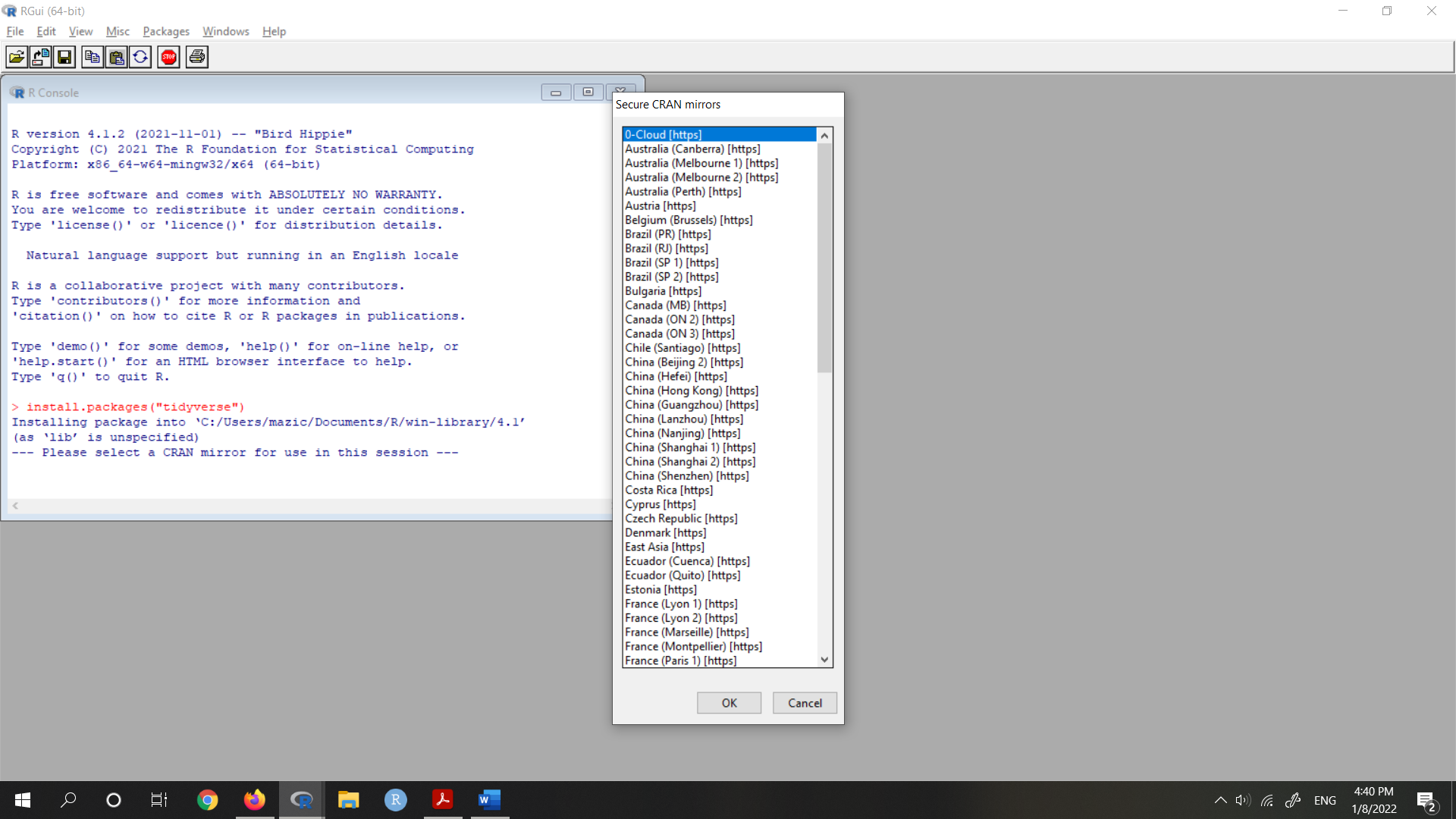


(2) Once the package is successfully installed, you will see the following highlighted message in R Console.


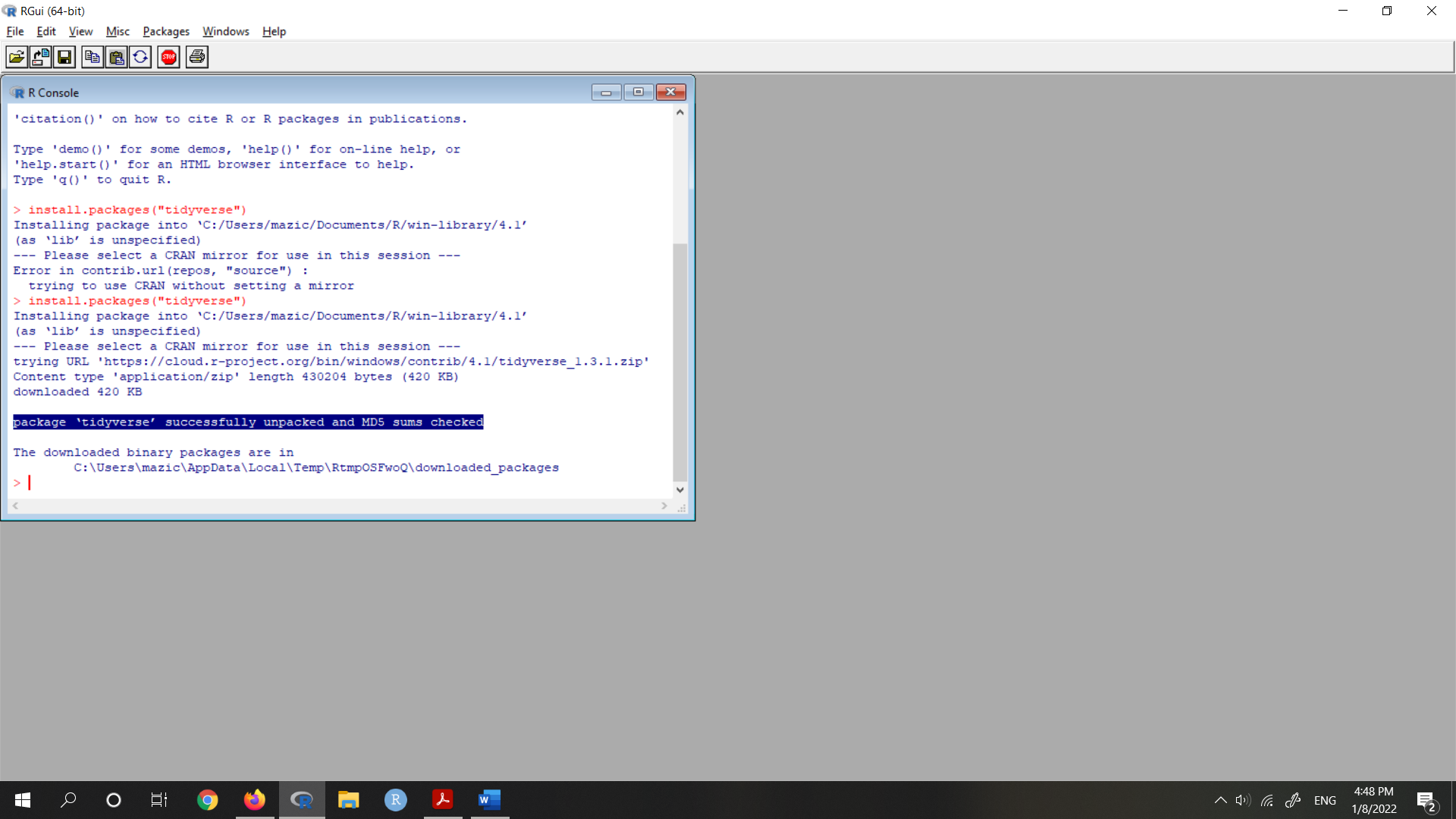


3. Demo and Instructions for Use

3.1 Find and open “Simulated_Table2.R” script.

(a) From the pull-down menu, click on “File” -> “Open script…”.


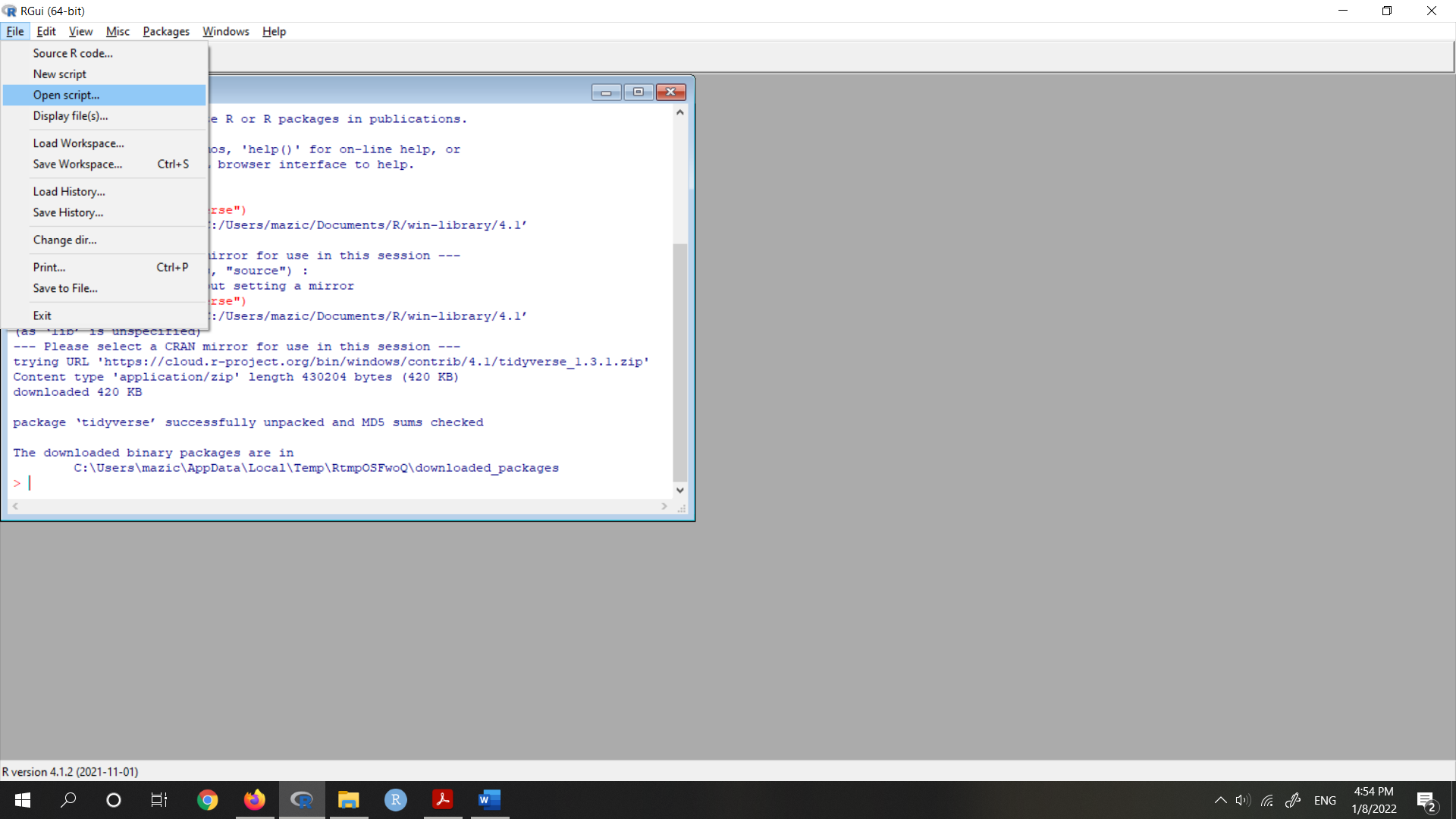


(2) In the new window, navigate to where the “Simulated_Table2.R” file is in your computer. In this case, it’s in the “\Desktop\NCOMMS-21-50698-T” folder. Select the file and click “Open”.


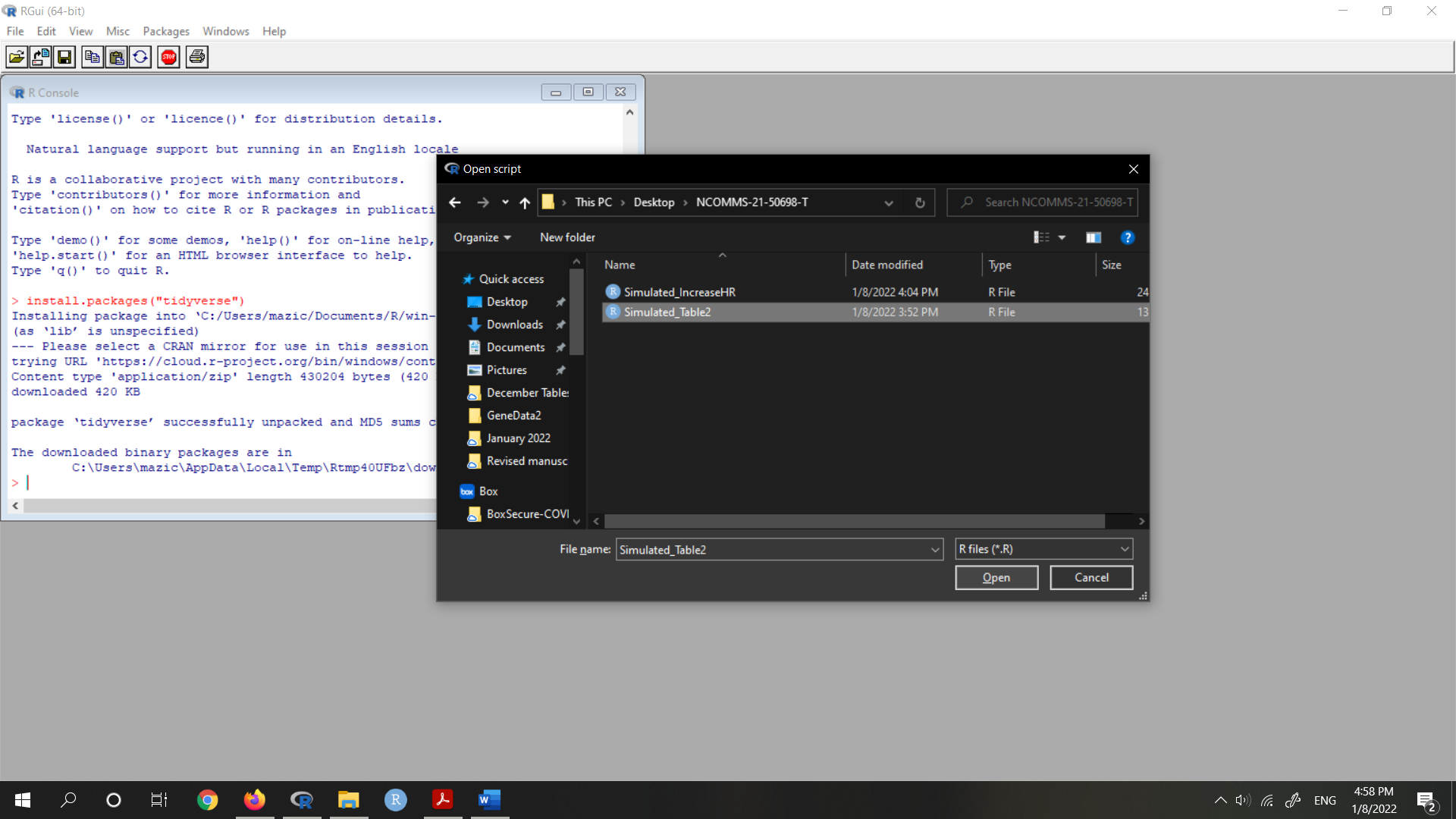


The R script will show up in a new window (R Editor).


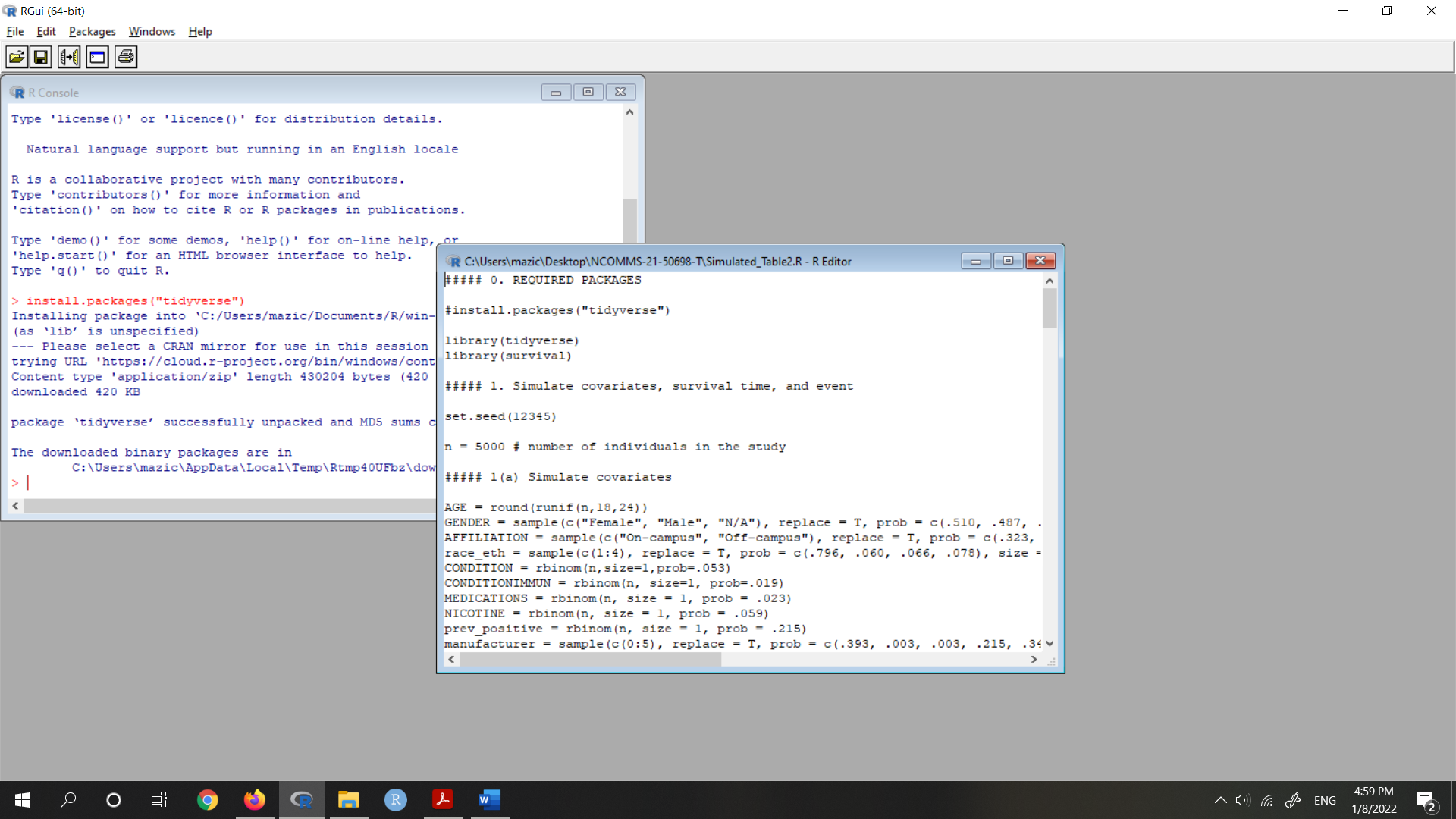


3.2 Run the “Simulated_Table2.R” script: While the R Editor is your main window (as seen above), hold “Ctrl” key and press “A”. This will select the entire script.


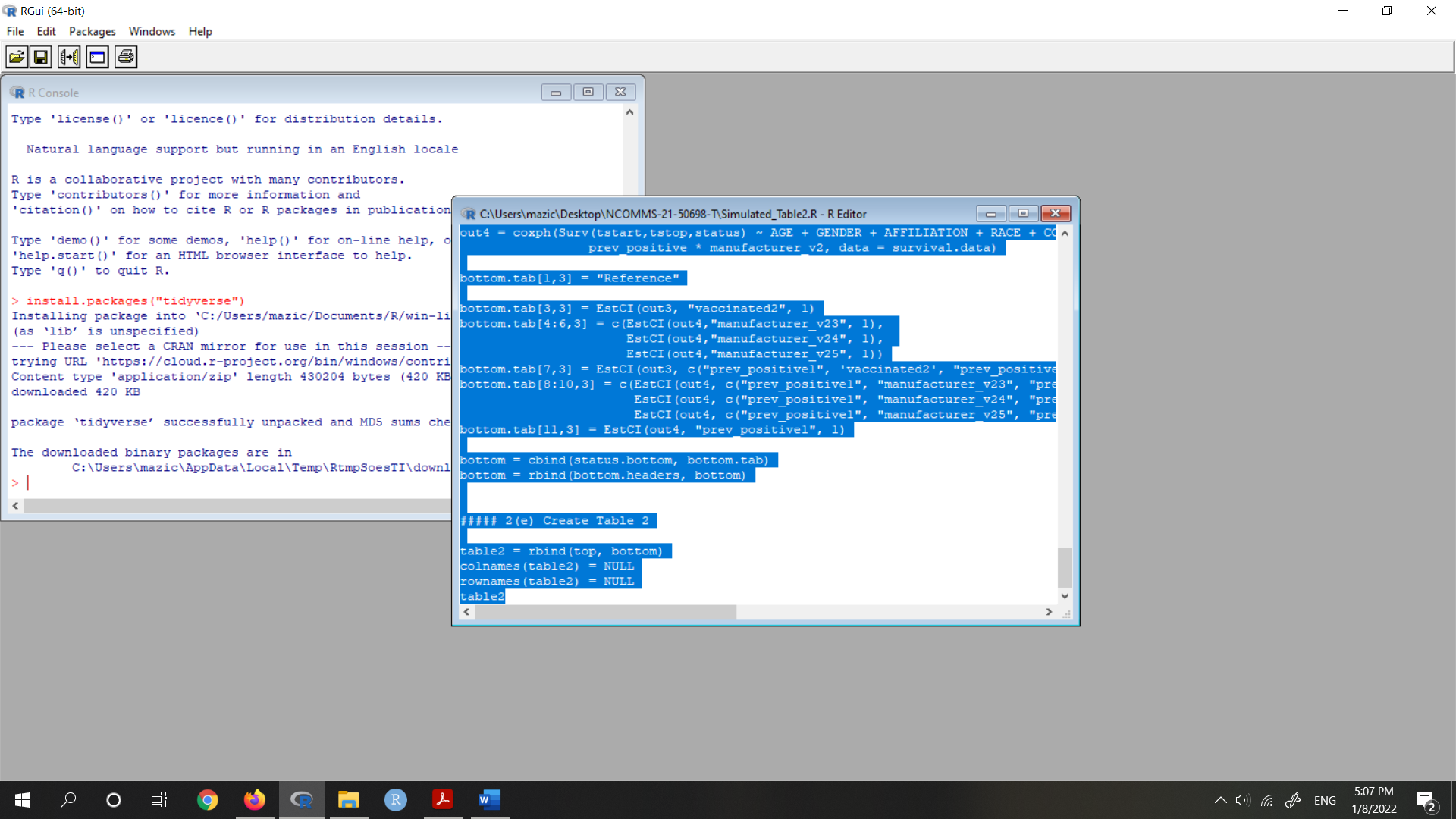


Then, hold the “Ctrl” key and press “R”. This will run the entire script. The following will be printed out in the R Console window.


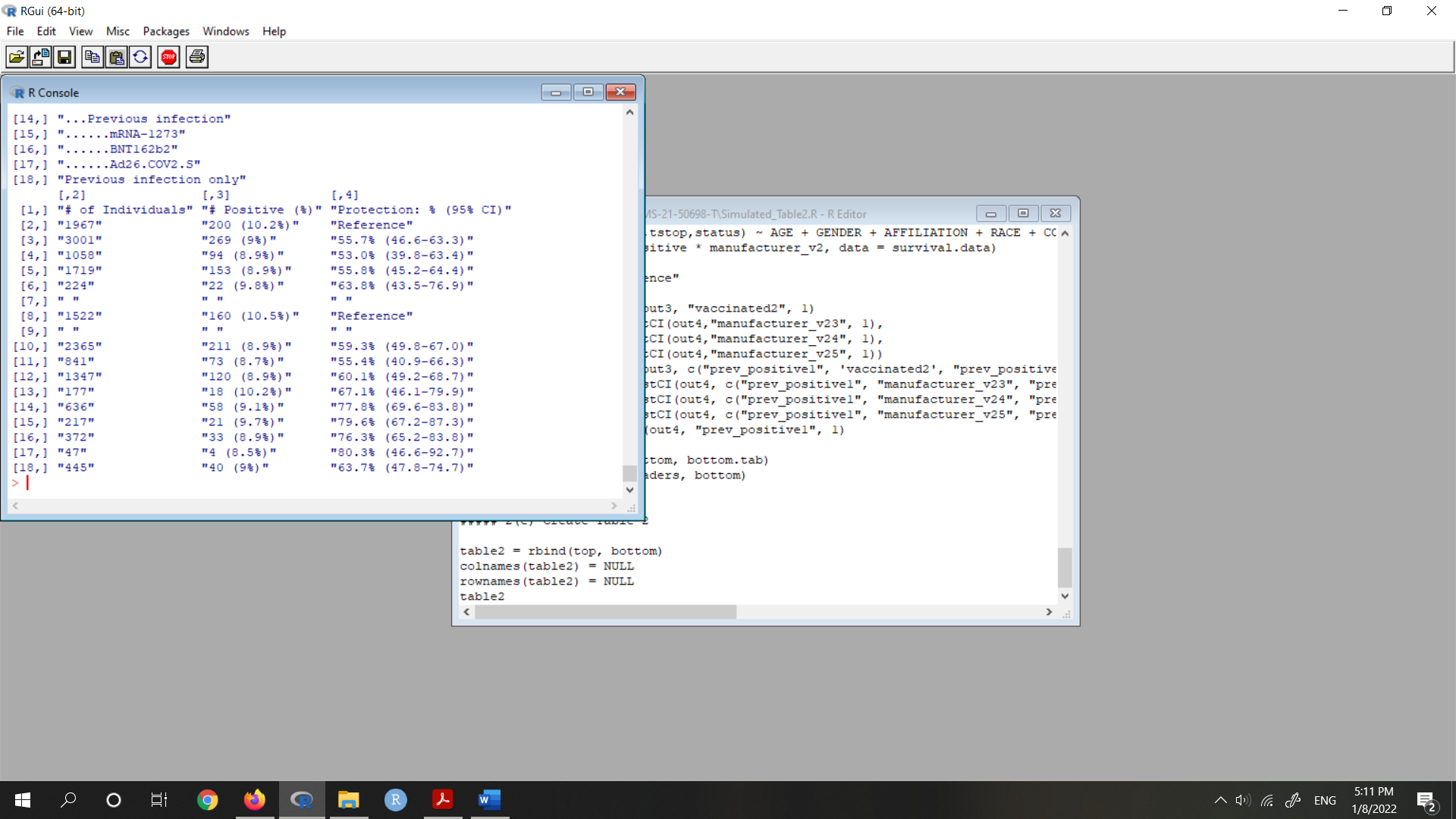


To have a better view of the entire output, maximize the R Console window. Then type “table2” in the Console and press Enter.


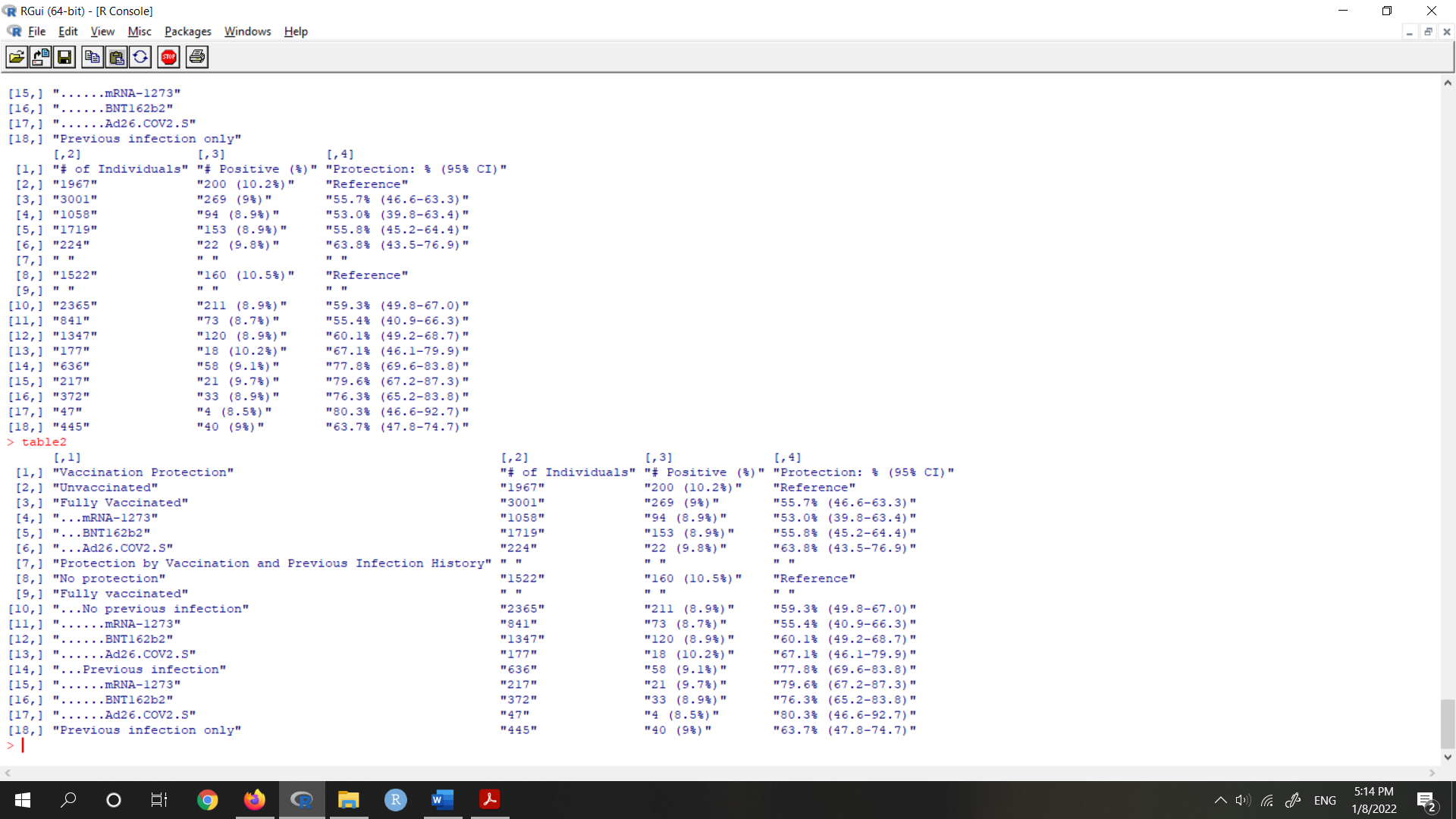


This reproduces Table 2 from the manuscript **based on a simulated data set.** The seed for generating random data was set at the beginning. The above is the precise result from running the R script. Running “Simulated_Table2.R” should take less than 30 seconds.

3.3 Repeat 3.1-3.2 to run the “Simulated_IncreasedHR.R” script. This file demonstrates the estimation of waning immunity of vaccination and previous infection in terms of the increase in the hazard ratio over time. As the “Simulated_Table2.R” script, this demonstration is also **based on a simulated data set.** The following “table3” is the precise result from running this R script. Running this R script should take less than 1 minute.


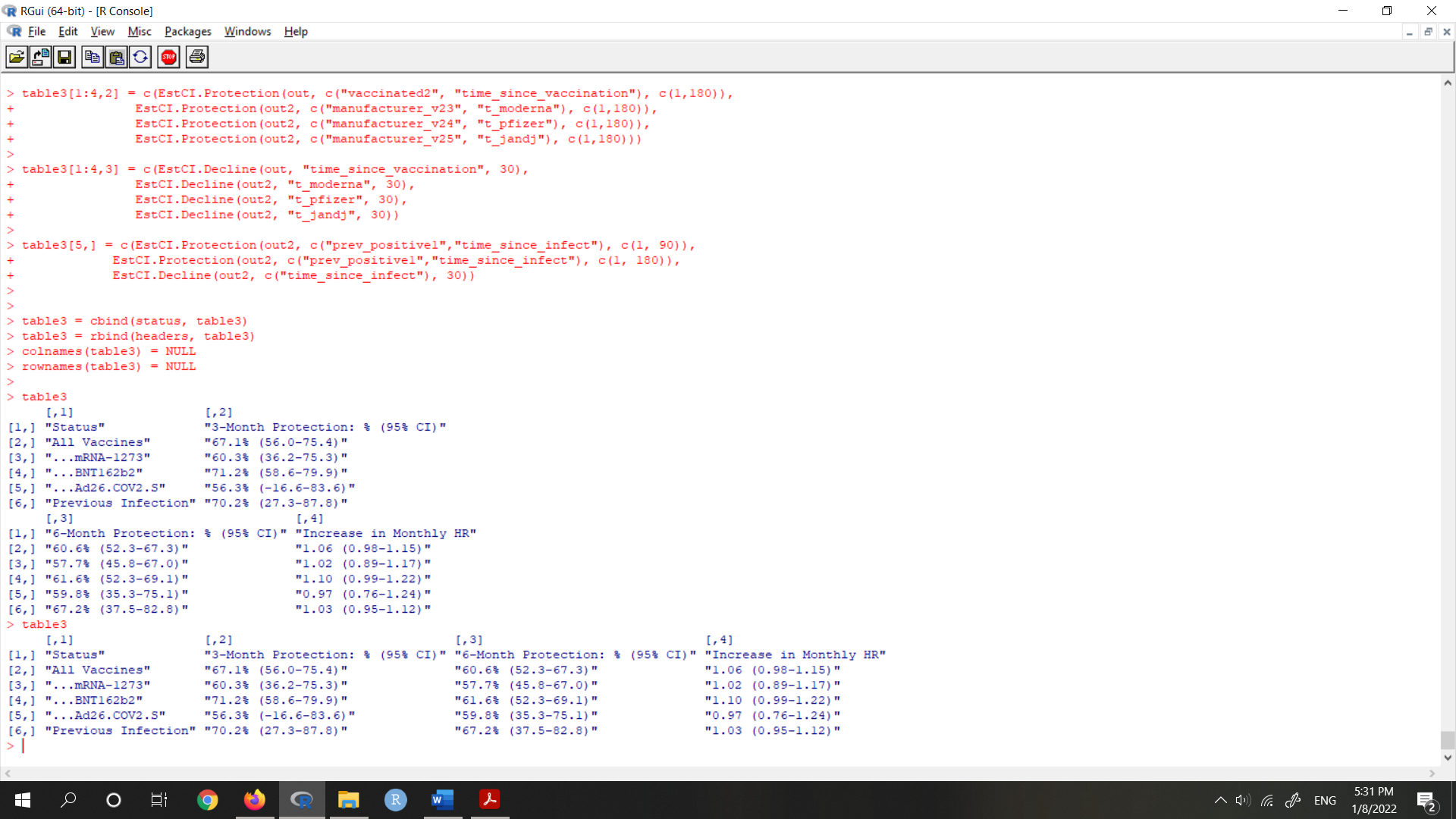

Supplement: Supplementary file 4 — Source Data [file 41467_2022_31469_MOESM4_ESM.zip › README.docx]
