# Supplementary figures and images for: Effectiveness and protection duration of Covid-19 vaccines and previous infection against any SARS-CoV-2 infection in young adults
Source: Nat Commun. 2022 Jul 8;13:3946. doi: 10.1038/s41467-022-31469-z (PMC9263799; doi:10.1038/s41467-022-31469-z)

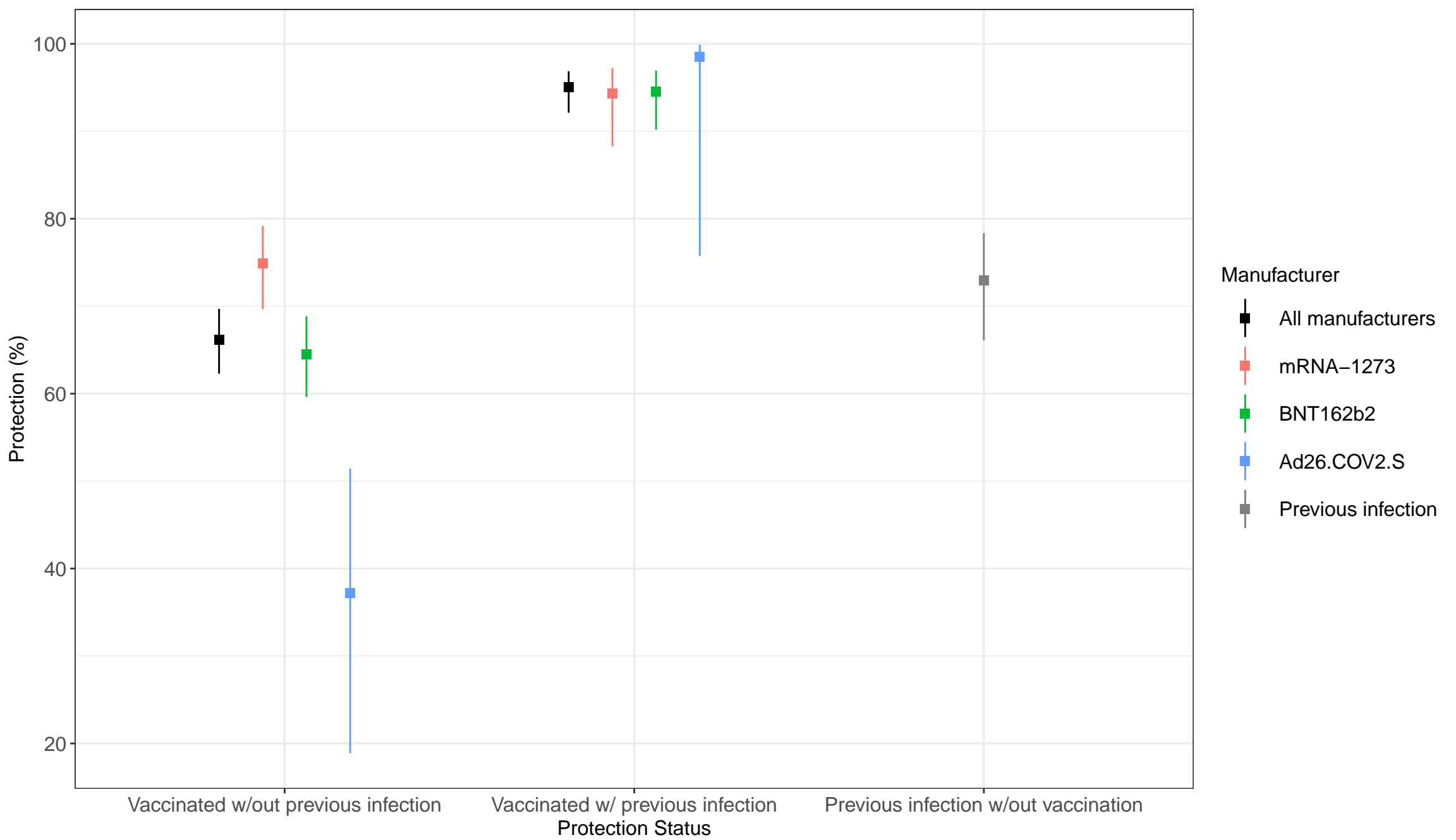

Supplement: Supplementary file 4 — Source Data [file 41467_2022_31469_MOESM4_ESM.zip › Figure1.pdf]

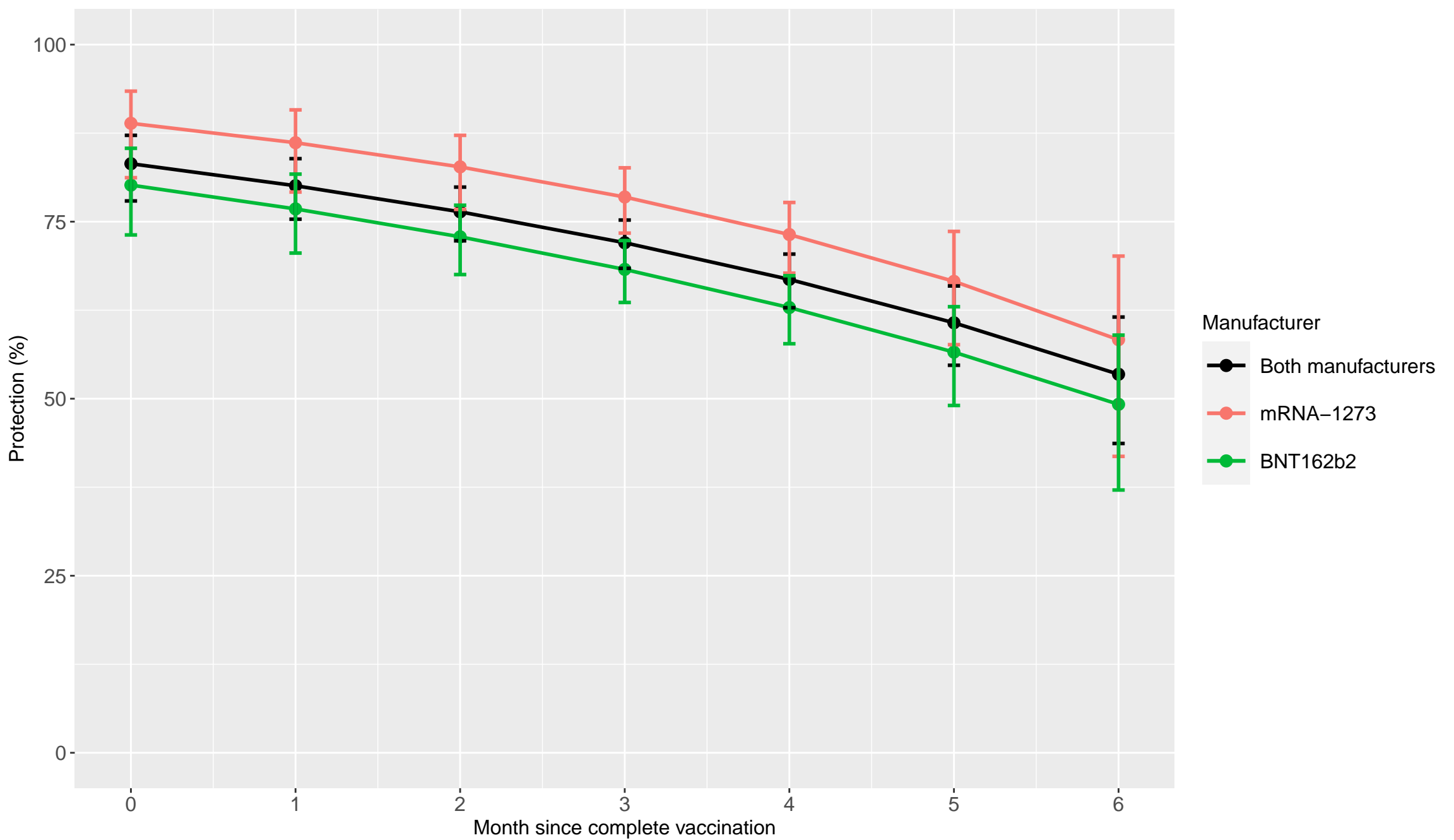

Supplement: Supplementary file 4 — Source Data [file 41467_2022_31469_MOESM4_ESM.zip › Figure2.pdf]

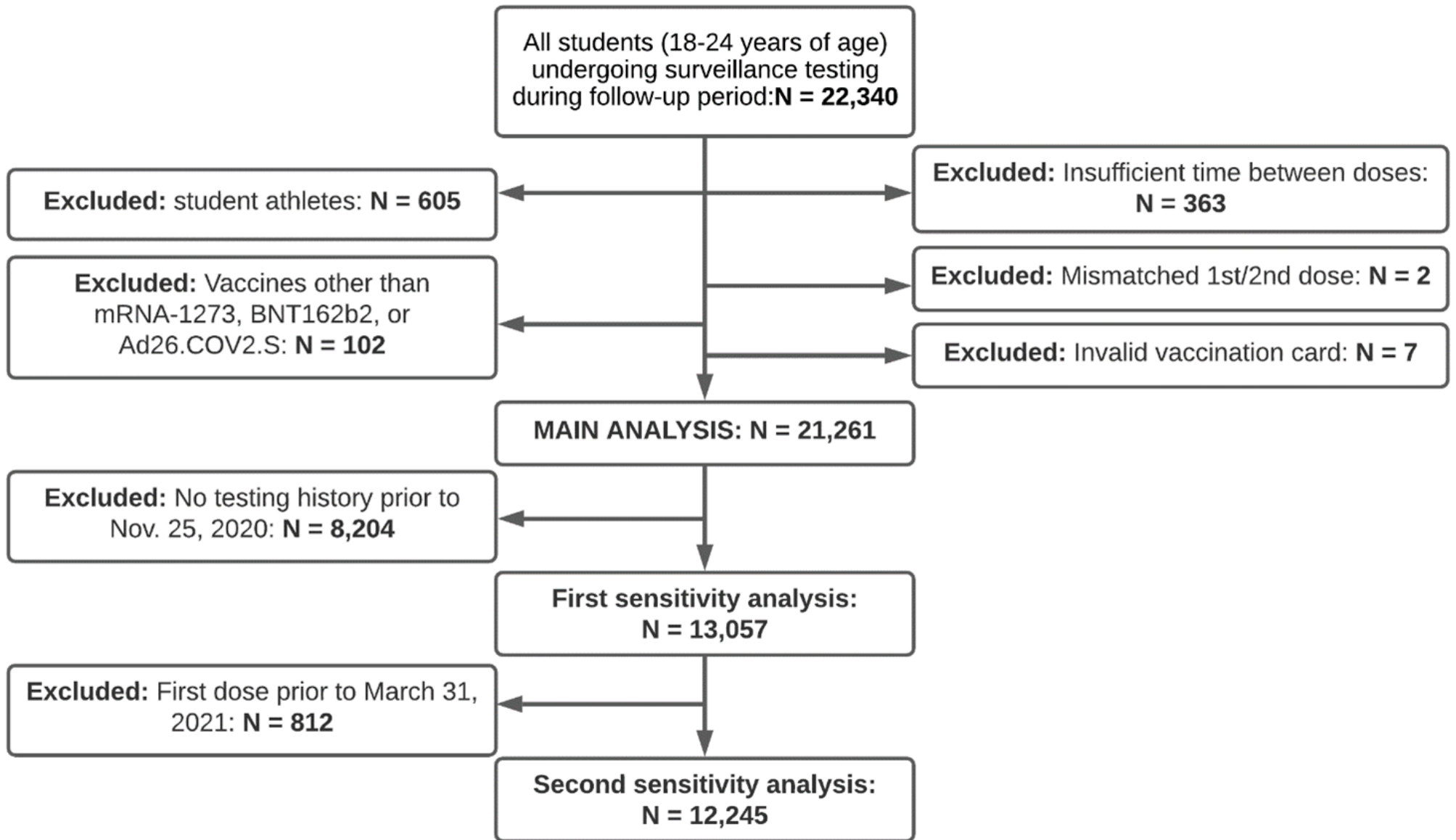

Supplement: Supplementary file 4 — Source Data [file 41467_2022_31469_MOESM4_ESM.zip › FigureS1.pdf]

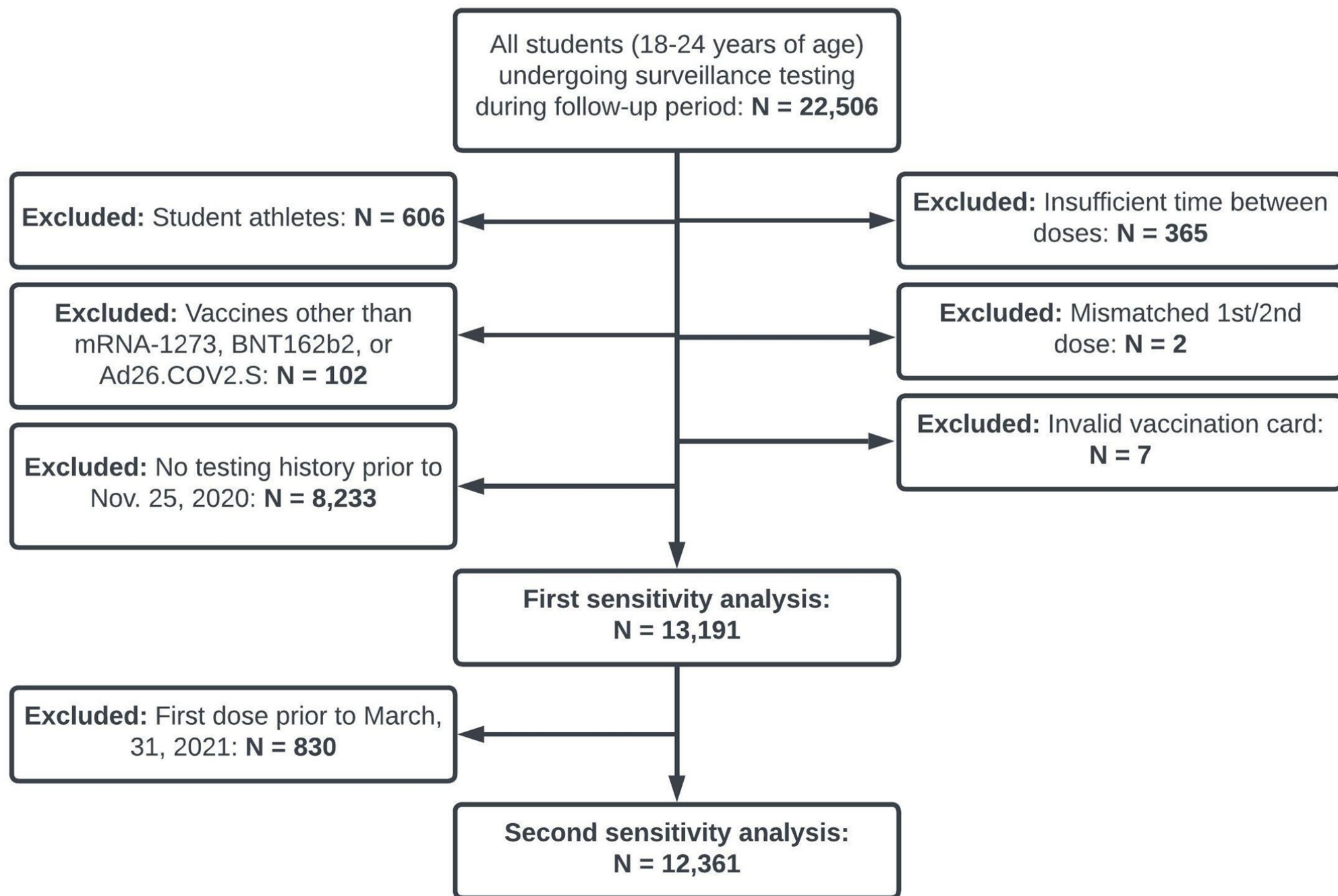

Supplement: Supplementary file 4 — Source Data [file 41467_2022_31469_MOESM4_ESM.zip › FigureS2.pdf]

Count

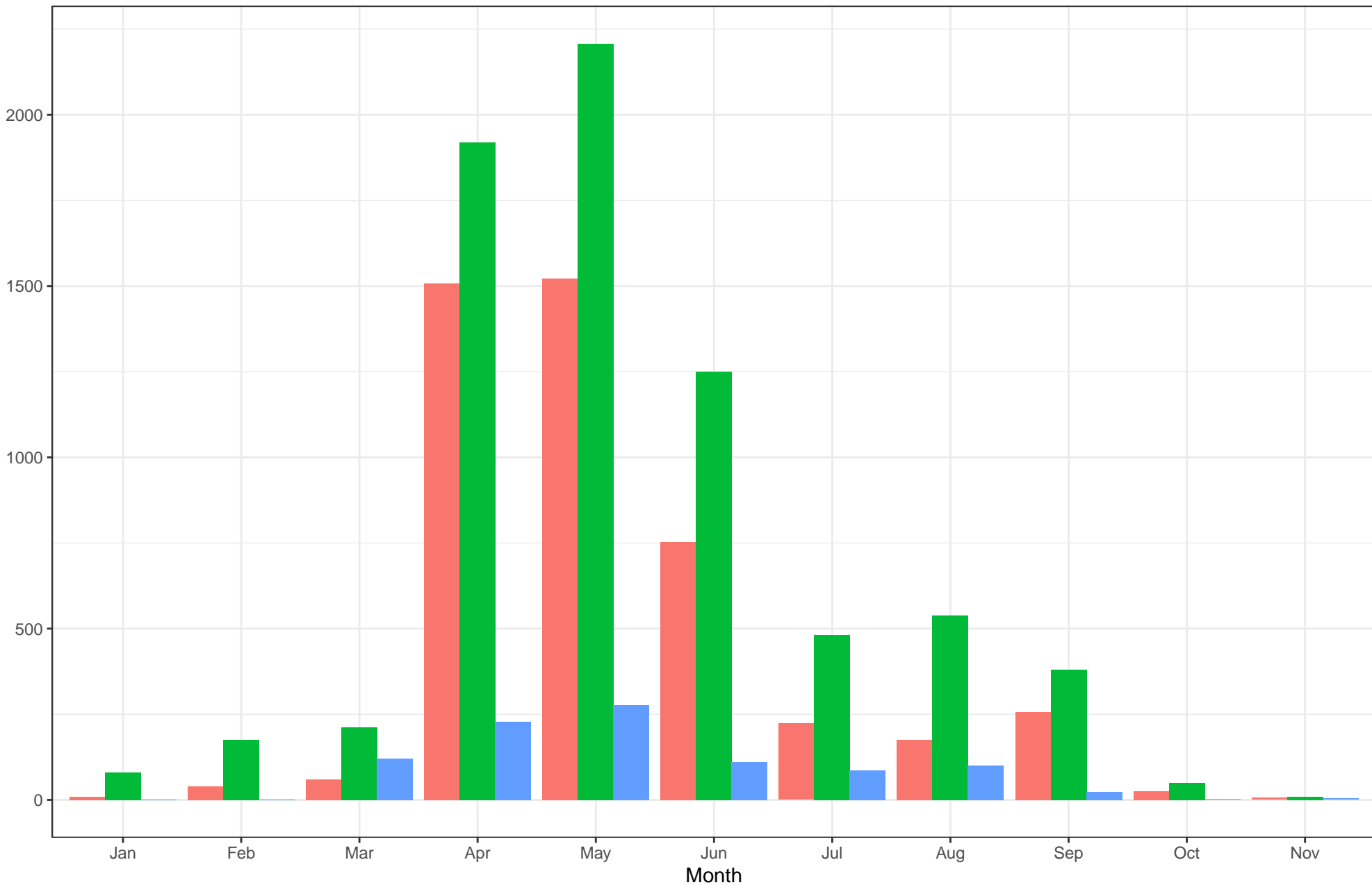

Manufacturer

- mRNA-1273
- BNT162b2
- Ad26.COV2.S

Supplement: Supplementary file 4 — Source Data [file 41467_2022_31469_MOESM4_ESM.zip › FigureS3.pdf]
